# Transcriptional regulatory logic of the diurnal cycle in the mouse liver

**DOI:** 10.1371/journal.pbio.2001069

**Published:** 2017-04-17

**Authors:** Jonathan Aryeh Sobel, Irina Krier, Teemu Andersin, Sunil Raghav, Donatella Canella, Federica Gilardi, Alexandra Styliani Kalantzi, Guillaume Rey, Benjamin Weger, Frédéric Gachon, Matteo Dal Peraro, Nouria Hernandez, Ueli Schibler, Bart Deplancke, Felix Naef, CycliX consortium

**Affiliations:** 1 The Institute of Bioengineering, School of Life Sciences, Ecole Polytechnique Fédérale de Lausanne, Lausanne, Switzerland; 2 Department of Molecular Biology, University of Geneva, Geneva, Switzerland; 3 Center for Integrative Genomics, Faculty of Biology and Medicine, University of Lausanne, Lausanne, Switzerland; 4 Department of Diabetes and Circadian Rhythms, Nestlé Institute of Health Sciences, Lausanne, Switzerland; 5 School of Life Sciences, Ecole Polytechnique Fédérale de Lausanne, Lausanne, Switzerland; Charité Universitätsmedizin Berlin, Germany

## Abstract

Many organisms exhibit temporal rhythms in gene expression that propel diurnal cycles in physiology. In the liver of mammals, these rhythms are controlled by transcription–translation feedback loops of the core circadian clock and by feeding–fasting cycles. To better understand the regulatory interplay between the circadian clock and feeding rhythms, we mapped DNase I hypersensitive sites (DHSs) in the mouse liver during a diurnal cycle. The intensity of DNase I cleavages cycled at a substantial fraction of all DHSs, suggesting that DHSs harbor regulatory elements that control rhythmic transcription. Using chromatin immunoprecipitation followed by DNA sequencing (ChIP-seq), we found that hypersensitivity cycled in phase with RNA polymerase II (Pol II) loading and H3K27ac histone marks. We then combined the DHSs with temporal Pol II profiles in wild-type (WT) and *Bmal1*^-/-^ livers to computationally identify transcription factors through which the core clock and feeding–fasting cycles control diurnal rhythms in transcription. While a similar number of mRNAs accumulated rhythmically in *Bmal1*^*-/-*^ compared to WT livers, the amplitudes in *Bmal1*^*-/-*^ were generally lower. The residual rhythms in *Bmal1*^-/-^ reflected transcriptional regulators mediating feeding–fasting responses as well as responses to rhythmic systemic signals. Finally, the analysis of DNase I cuts at nucleotide resolution showed dynamically changing footprints consistent with dynamic binding of CLOCK:BMAL1 complexes. Structural modeling suggested that these footprints are driven by a transient heterotetramer binding configuration at peak activity. Together, our temporal DNase I mappings allowed us to decipher the global regulation of diurnal transcription rhythms in the mouse liver.

## Author summary

The molecular circadian clock is conserved from cyanobacteria to mammals and is believed to align behavioral and biochemical processes with the day’s 24-h diurnal cycle. How the circadian clock and feeding rhythm transcriptionally interact and what the contribution is of cis-regulatory modules to this interconnection has not yet been fully elucidated. To address these questions, we explored the dynamics of accessible regions, histone modifications, and RNA polymerase II loading on the scale of hours in the liver of wild-type (normal) mice and mice that are mutant for the clock master regulator BMAL1. This allowed us to uncover circadian clock–and feeding-dependent gene regulatory networks. Furthermore, we dissected the chromatin accessibility around BMAL1-binding sites at base pair resolution. This enabled us to develop a new DNA-binding model for BMAL1/CLOCK involving the formation of a heterotetramer configuration at times of peak activity. Overall, these temporal profiles provide insight into the global regulation of daily transcription rhythms in the mouse liver.

## Introduction

Circadian clocks provide mammals with cell-autonomous and organ-based metronomes that relay diurnal environmental cues to temporal gene expression programs [1,2]. In particular, diurnal rhythms in mRNA transcription result from the combined actions of the autonomous circadian oscillator, systemic signals, and other temporal cues such as feeding–fasting cycles [3–6]. While it is commonly assumed that around 10% of genes exhibit cyclic mRNA levels in the liver [7], this number increases to nearly 50% when only considering specifically liver-expressed genes [8]. Moreover, these mRNA rhythms cover a continuum of peak times [9,10]. Although mRNAs can also rhythmically accumulate because of posttranscriptional regulation [6,11–14], it is of interest to obtain a more comprehensive view on transcriptional regulators and mechanisms underlying time-specific diurnal transcription. In a light–dark (LD) cycle, two main waves of transcription are found: one during the day (at around zeitgeber time [ZT]10) and the other towards the end of the night (around ZT20), accompanied by dynamic chromatin state modifications [6,11,12].

Current models of time-specific transcription in the liver involve the core clock transcription factors (TFs) CLOCK:BMAL1 that activate transcription maximally at ZT6 [15–17] as well as the nuclear receptors RORs and REV-ERBs, whose targets are maximally transcribed around ZT20 [18,19]. Rhythmically active TFs also include clock-controlled outputs, notably the PAR-bZIP proteins (DBP, TEF, HLF), which are maximally active near ZT12 [16,20]. Furthermore, diurnally fluctuating systemic signals may drive rhythmic TF activities: for example, heat shock transcription factor 1 (HSF1) shuttles to the nucleus and activates transcription at ZT14 [21,22], and similarly, SRF shows activity at the night–day transition [23]. Moreover, regulators controlled by feeding–fasting cycles include forkhead box (FOX)O TFs that are active during the day, CREB/ATF family members at the light–dark transition, and SREBP during the night [5,24]. Finally, the glucocorticoid receptor (GR) signals the onset of behavioral activity (light–dark transition) [25].

Frequently, these factors act by binding to sequence-specific DNA elements located in the vicinity of gene promoters [26,27]; however, less is known about more distally located enhancer regulatory elements involved in diurnal transcriptional control. To start identifying such regulatory elements, recent maps of the activity-related chromatin mark H3K27ac [6], as well as enhancer RNAs (eRNAs) [28], were established. These studies identified thousands of putative enhancers with a broad range of peak activity times, which were associated with distinct DNA regulatory motifs and TF-binding patterns.

Here, we used genome-wide DNase I hypersensitivity mapping [29] to further identify temporally active transcriptional regulatory elements. In the context of the circadian clock, DNase I hypersensitive site (DHS) mapping was first applied to study regulation of the *Dbp* gene in the mouse liver, which led to the identification of several DHSs located in 5′-flanking and intronic regions [30]. Several of those regions showed diurnal rhythms in hypersensitivity with amplitudes as large as 3-fold, which prompted us to generate a temporally resolved and genome-wide DNase I hypersensitivity map [31,32]. We detected around 65,000 DHSs in the mouse liver, of which 8% cycled. Moreover, such cycling hypersensitivity occurred in phase with RNA polymerase II (Pol II) loadings and H3K27ac histone marks, suggesting that DHSs harbor regulatory elements controlling rhythmic transcription. Analysis of wild-type (WT) and circadian clock–deficient *Bmal1*^*-/-*^ animals enabled us to identify transcription regulators with activities at specific times of the day and to explore how feeding rhythms affect oscillatory activation of transcription in the absence of a functional circadian clock. By contrasting DHS sites in WT and *Bmal1*^*-/-*^ animals, we uncovered that BMAL1 has limited but specific impact on DNA accessibility in regulatory regions. Finally, because DNase I hypersensitivity mapping leaves characteristic footprints at sites of bound TFs, we could study the temporal dynamics of TF complexes bound to DNA. This allowed us to propose a temporal DNA-binding mode for the CLOCK:BMAL1 heterotetramer complex.

## Results

### DNase I hypersensitive site mapping during diurnal cycles in the mouse liver

**Fig 1:**
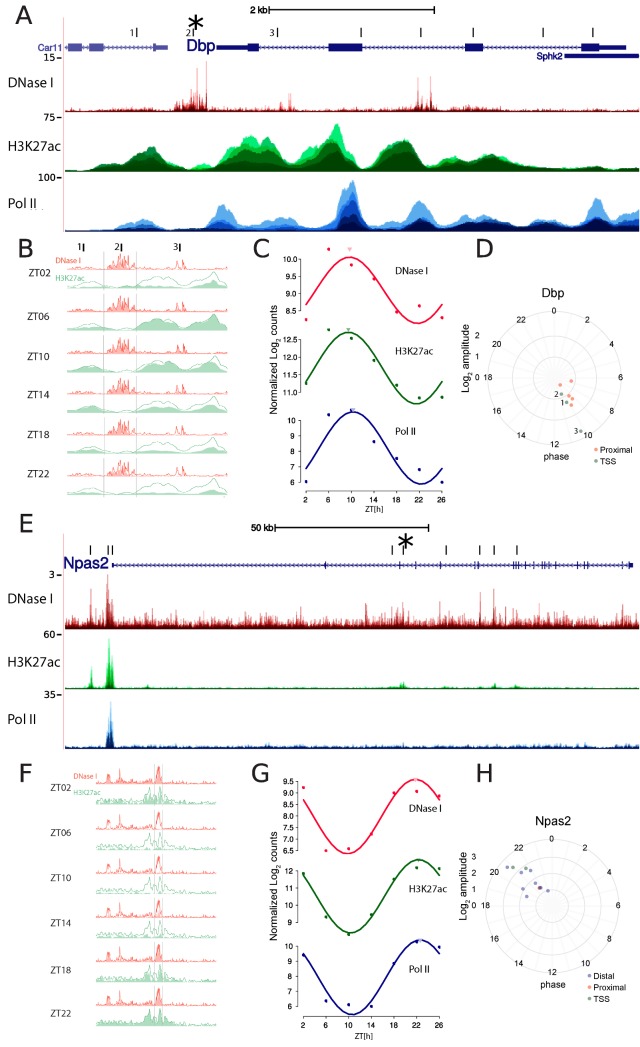
DNase I hypersensitivity is rhythmic during diurnal cycles in mouse liver. A. DNase I hypersensitivity, RNA polymerase II (Pol II) density, and H3K27ac enrichment at the *Dbp* locus. The DNase I track shows the frequency at which nucleotide-resolved DNase I cuts, while H3K27ac and Pol II chromatin immunoprecipitation followed by DNA sequencing (ChIP-seq) signals are smoothed over 100 bp. All time points are overlaid. The center of each DNase I hypersensitive site (DHS)-enriched region is indicated by vertical ticks (three sites near the TSS are numbered). B. Zoom-in around the transcription start site (TSS) of *Dbp* (the three TSSs in A are marked) reveals DNase I cuts in between H3K27ac-marked nucleosomes. Both DNase I and H3K27ac signals are maximal at ZT6–ZT10 and minimal at ZT22, consistent with BMAL1-mediated activation of *Dbp* transcription (absolute signal is highest for site 2, while amplitude is highest for site 3; see panel D). The red and green lines (identical in all subgraphs) show the max signal over the time points at each position and serve as a guide to the eye. C. Quantification of read counts (in log_2_ units) for DNase I cuts (in windows of ±300 bp) and Pol II and H3K27Ac ChIP-seq data (in windows of ±1,000 bp) centered on the *Dbp* TSS using cosine fits. Cosine fits show a common estimated peak time around ZT10 (marked by the inverted triangles). Peak-to-trough amplitudes are about 16-fold for Pol II and approximately 4-fold for both DNase I and H3K27ac. D. Phases and amplitudes of all DHS sites located in the neighborhood of the *Dbp* gene (nearest TSS association according to annotation). Distances from the center of the plot indicate fitted log_2_ amplitudes, and angles (clockwise from ZT0) indicate peak times. We observed that all regions oscillate around a common phase of ZT10. Of the three sites near the TSS (numbered 1–3), site 3 has the highest amplitude. E–H. Idem as A–D but for *Npas2*, which has an opposite phase to *Dbp* (i.e., *Npas2* peaks near ZT22). Oscillatory amplitudes are generally larger for *Npas2* compared to *Dbp*. G shows quantification of the signal at the TSS as in panel C.

To identify DNA regulatory elements controlling diurnal transcriptional rhythms in the mouse liver, we mapped DHSs every 4 h during a full LD cycle. Specifically, C57BL/6 male mice were kept in standard 12-h light–12-h dark cycles, and four animals were euthanized every 4 h for 1 d followed by liver dissection (Materials and methods). DNase I hypersensitivity libraries were produced, sequenced, and mapped to the mouse genome using standard methods (Materials and methods). To monitor transcription activity in the same conditions, we generated chromatin immunoprecipitation followed by DNA sequencing (ChIP-seq) samples for the histone modification H3K27ac (marking active regulatory elements [33]) and resequenced previous total Pol II ChiP-seq libraries [12] at increased coverage (Materials and methods and S1 Table). Circadian clock outputs result in the rhythmic transcriptional activation of hundreds of genes, notably through binding of CLOCK:BMAL1 heterodimers [16,17]. To validate our assays, we therefore examined the known circadian output gene *Dbp* (S1 Movie), maximally transcribed at ZT8 [30], to determine whether cutting frequency at DHSs exhibited diurnal variation. We detected several DHSs in the vicinity of *Dbp*, with high intensity and narrow signals surrounded by low noise levels (Fig 1A). These DHSs near *Dbp* coincided well with regions identified using classical DHS mapping [30] and overlapped with BMAL1 ChIP-seq regions [17] (S1 Fig). As exemplified by DHSs near the transcription start site (TSS) of *Dbp*, we observed that DHSs were located in regions with lower H3K27ac signals in between H3K27ac-enriched islands, which is suggestive of TF-induced nucleosome displacement [34–36] (Fig 1B). The DNase I hypersensitivity changed diurnally, notably at the TSS (Fig 1C), where the oscillations in DNase I hypersensitivity, Pol II, and H3K27ac peaked in sync at ZT10 (Fig 1B). Moreover, all DHSs within 15 kb of the *Dbp* TSS displayed oscillations of varying amplitudes (maximally about 7-fold), consistent with [30], but all with the same phase as the TSS (Fig 1D), suggesting regulatory relationships between these regions and gene transcription.

We next analyzed the *Npas2* gene (S2 Movie), another known clock target [37]. *Npas2* is a target of RORs and peaks in the late nighttime around ZT22 [38]. We detected several DHSs along the transcribed region of *Npas2* (Fig 1E), including proximal (defined as 1–10kb from a TSS) and distal (defined as >10kb from a TSS) sites. The distal sites displayed high-amplitude oscillations of DNase I signals and H3K27ac (Fig 1F). Normalized signals at the *Npas2* TSS also peaked at the expected phase, with maximal signal at ZT22 for all three marks studied (Fig 1G). Finally, all DHSs associated with *Npas2* (those having *Npas2* as their closest TSS), including numerous distal regions, likewise cycled with phases around ZT22, some with amplitudes higher than 10-fold (Fig 1H). The examples of the *Dbp* and *Npas2* loci suggest that our genome-wide study detected DHSs with high resolution and that the temporal patterns of DNase I cuts reflected diurnal activities of these elements.

### Identification of regulatory elements and transcription factor footprints in mouse liver DHSs

To comprehensively map putative regulatory elements across the genome, we merged our DNase I hypersensitivity time points and performed peak finding (Materials and methods). This revealed 62,418 DHS sites, covering around 2% of the mappable genome (considering a width of 600 bp for each DHS site), which is comparable to previous studies across mouse tissues [39] (all sites and associated signals in S2 Table). Because we aimed at associating DHSs with nearby genes to infer regulatory relationships, we first discarded transcripts from Ensembl annotations that were not expressed in our samples. For this, we used histone modifications, Pol II profiles, and now also DNase I signals at transcription start and end sites of annotated transcripts to train a supervised learning method (support vector machine) that distinguishes expressed (active) from nonexpressed genes, similar to our previous work [12] (Materials and methods). To infer putative regulatory relationships, we then annotated each DHS to the nearest active TSS. Distances between DHSs and TSSs followed a bimodal distribution, with a first mode around 100 bp from the TSSs and a second 10 kb from the TSS (S2A Fig). Consistent with previous reports [40,41], one-third of our DHSs were found within 1 kb of a TSS, while almost half were located more than 10 kb from a TSS (S2B Fig). At TSSs, the genomic distributions of DNase I cuts, Pol II, and H3K27ac signals (centered on TSSs) were consistent with accessibility of DNA being determined by nucleosome displacement and Pol II complex assembly (S2C Fig) [42]. At distal DHSs, profiles of H3K27ac showed a dip in the peak center, consistent with occupation by TFs and nucleosome displacement (S2D Fig), while the weaker Pol II signals could reflect distal assembly of the transcriptional complex [43] or interactions between enhancer regions and the TSS through DNA looping [44,45].

**Fig 2:**
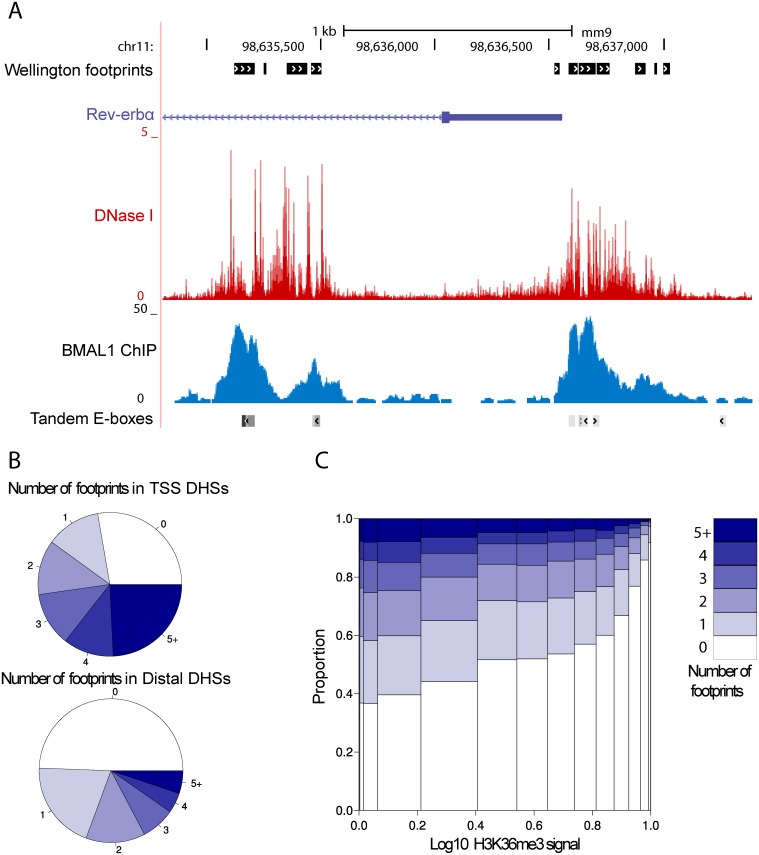
Location-dependent footprint characteristics of DNase I Hypersensitive Sites (DHSs). A. Visualization of DNase I signal (red) around the *Rev-erbα* promoter with the footprints (detected by Wellington) annotated in black, on top. This region contains BMAL1-binding sites (blue) with E-box motifs, annotated on the bottom line, which is marked by a characteristic footprint. The DNase I cleavage pattern is lower at the binding site, reflecting protection of the DNA from digestion, whereas high signals are observed on the edges of the binding site. B. Number of footprints within DHSs (±300 bp around the peak center). TSS regions contain more footprints on average. More than half of distal regions contain a footprint. C. Number of footprints detected in DHSs in function of (relative) H3K36me3 signal [12].

To determine whether DHSs reflected DNA-bound transcription regulators, we searched for short windows protected from cleavage (or footprints) [46] within a ±300 bp window around the center of each DHS. This identified previously reported footprints, as illustrated for the well-characterized promoter of the *Albumin (Alb)* gene [47] (S2E Fig). In the promoter region of *Rev-erbα (Nr1d1)*, the detected footprints coincided with E-boxes and high BMAL1 ChIP-seq signals (Fig 2A). Overall, the majority (70%) of DHSs within 1 kb of a TSS contained at least one footprint, while this proportion dropped to one-half for proximal (defined as DHSs within 1–10kb of a TSS) or distal (>10 kb of a TSS) DHSs (Fig 2B). Since transcribed DNA is known to be DNase I sensitive [48], the DHSs without footprints might reflect transcription. To test this, we analyzed the number of footprints in DHSs outside of promoter regions and further marked with H3K36me3, a mark coinciding with transcribed gene bodies [12,49]. Indeed, DNase I hypersensitive regions without footprints were frequently (90%) linked with highly transcribed genes (Fig 2C). Thus, DHSs at TSS seemed to contain more footprints than distal DHSs, and transcription elongation explains why some DNase I hypersensitive regions did not exhibit a footprint.

### TSSs and distal regulatory elements display 24-h oscillations in DNase I hypersensitivity in sync with Pol II and H3K27ac enrichment

**Fig 3:**
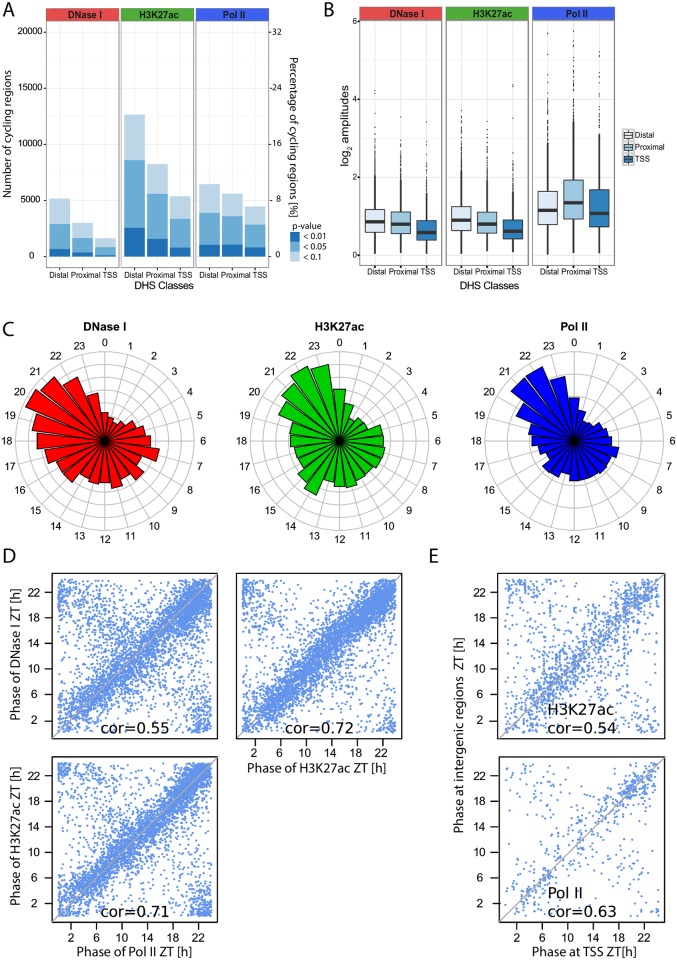
Genome-wide rhythms in DNase I signals are synchronous with RNA Polymerase II (Pol II) transcription and histone acetylation. A. Number of DNase I hypersensitive sites (DHSs) with statistically significant cycling DNase I signals (left), H3K27ac signals (middle), or Pol II signals (right) at three different thresholds (*p* < 0.1, *p* < 0.05 and *p* < 0.01, harmonic regression), partitioned according to their genomic location: TSS (1 kb), proximal (1–10 kb from TSS), or distal (>10 kb from TSS). B. Comparison of log_2_ amplitudes for DHSs in each class (TSS, proximal, and distal) and in each signal (Pol II, H3K27ac, and DNase I). We selected 4,606 sites (FDR < 0.05, Fisher's combined test). Higher amplitudes were observed in distal and proximal regions compared to TSSs (*p* < 2.2*10^−16^, *t* test). In addition, Pol II loadings showed higher peak-to-trough ratios than the two other signals. C. Circular histograms representing the distributions of phases for each mark at DHSs selected as in B. D. Comparisons of peak times between DNase I, Pol II, and H3K27ac at DHSs (DHSs selected with *p* < 0.05, Fisher’s combined test), diagonals are indicated in gray. Values of circular correlations are indicated (*p* < 10^−10^, circular correlation). E. Relationships of peak times between DHSs in intergenic regions with their nearest TSS (pairs selected with FDR < 0.1, Fisher’s combined test). We found 1,611 and 630 significant pairs for H3K27ac and Pol II signals, respectively.

We next studied whether DNase I hypersensitivity, Pol II density, and H3K27ac quantified at the identified DHSs displayed diurnal rhythms using harmonic regression (Materials and methods). The number of cyclic regions identified at different significance thresholds clearly indicated that Pol II and H3K27ac oscillated at a larger number of DHSs compared to the DNase I signal itself, both for proximal and more distal DHSs (Fig 3A). To select rhythmically active regions, we assessed the combined rhythms of the three marks at each DHS as previously described using Fisher's combined test [12,50], which yielded 4,606 DHSs (7.3%, false discovery rate [FDR] < 0.05). For all three signals, the amplitude of the oscillations was larger at distal DHSs (the median peak-to-trough amplitude was 2-fold for DNase I and H3K27ac and higher for Pol II) compared to TSSs, and Pol II had larger amplitudes than either DNase I or H3K27ac (Fig 3B). Moreover, the peak times of the oscillations in DNase I signals were, except for some small deviations, similarly distributed as peak times in gene transcription and H3K27ac [6,11,12], with a weak evening peak around ZT10 and a marked late-night peak around ZT22 (Fig 3C). We next considered the relationships of peak times in the DNase I, Pol II, and H3K27ac rhythms. It is known that many chromatin marks exhibit diurnal rhythms that are tied to transcription [6,11,12,16], and similarly, enhancer RNAs (eRNAs) were shown to be transcribed in sync with their cognate transcripts [28]. We observed that DNase I cuts, Pol II, and H3K27ac displayed synchronous oscillations at DHSs (Fig 3D). Such relationships were maintained after removing DNase I–sensitive regions situated in the transcribed region of active genes (S3 Fig), indicating that this phenomenon was not a mere reflection of transcription [51]. To test whether the signals measured at DHSs near TSSs were temporally correlated with those at proximal or distal DHSs, we examined pairs of oscillating DHSs (FDR < 0.1, Fisher’s combined test), of which one was located near a TSS (<1 kb) and the other in an intergenic region positioned at least 2 kb and at most 20 kb from any TSS. While no pair reached statistical significance for DNase I signals (at the level of FDR < 0.1), probably reflecting that DNase I signals are noisier than the two other marks, we found 1,611 pairs oscillating for H3K27ac and 630 for Pol II. The two peak times were highly correlated, with differences within 1 h (Fig 3E), suggestive of enhancer–TSS interactions [40].

### Computational analysis identifies transcription factors through which the circadian clock and feeding–fasting cycles control diurnal gene expression

To understand how the circadian clock and the feeding–fasting cycle control diurnal gene expression in the liver, we studied mRNA expression (from [24]) and Pol II loading at TSSs in WT and *Bmal1*^-/-^ mice subject to the same night-restricted feeding regimen (S4 Fig). First, we observed that a similar number of genes oscillated in the WT and *Bmal1*^*-/-*^ genotypes; however, these genes had an overlap of about 30% for Pol II and 50% for mRNA (when we selected oscillations with *p* < 0.05). This indicates that genes with a diurnal expression differ between WT and *Bmal1*^-/-^ mice (S4A Fig). While such comparisons depend on cutoffs, stratifying by peak-to-trough amplitudes clearly showed that high-amplitude rhythms are more abundant in WT as compared to *Bmal1*^-/-^ mice (S4B Fig) and that this was more pronounced for mRNA than for Pol II loading at TSSs. For example, we found 12 genes with more than 10-fold mRNA amplitudes in WT and only 3 in *Bmal1*^*-/-*^ mice. Genes with Pol II or mRNA rhythms in both genotypes showed highly correlated phases, with a tendency for a slight average delay (~1 h in Pol II and less in mRNA) in the absence of a circadian clock (S4C and S4D Fig). Functional annotation using KEGG and Reactome pathways and comparison between mRNA rhythms in WT and *Bmal1*^-/-^ animals revealed that genes annotated for circadian rhythm, bile secretion, steroid metabolism, ribosome biogenesis, and SREBP signaling were enriched in the WT condition at the expected times of the day (S5A Fig). In *Bmal1*^-/-^ mice, circadian rhythms were obviously absent, but interestingly, most of the other functions were still oscillating, notably SREBP and ChREBP signaling. In general, the lipid related processes even had higher amplitudes in the *Bmal1*^-/-^ animals (S3 Table, S5A and S5B Fig). These findings are consistent with our observation that genes bound by BMAL1 or CLOCK cycle with much greater amplitudes in the WT compared to *Bmal1*^*-/-*^ mice. Conversely, genes bound by nutrient-responsive and systemic TFs (CREB, SREBP, HSF1) exhibited either much smaller differences (CREB) between these two conditions or no difference (SREBP, HSF1) (S6A Fig).

**Fig 4:**
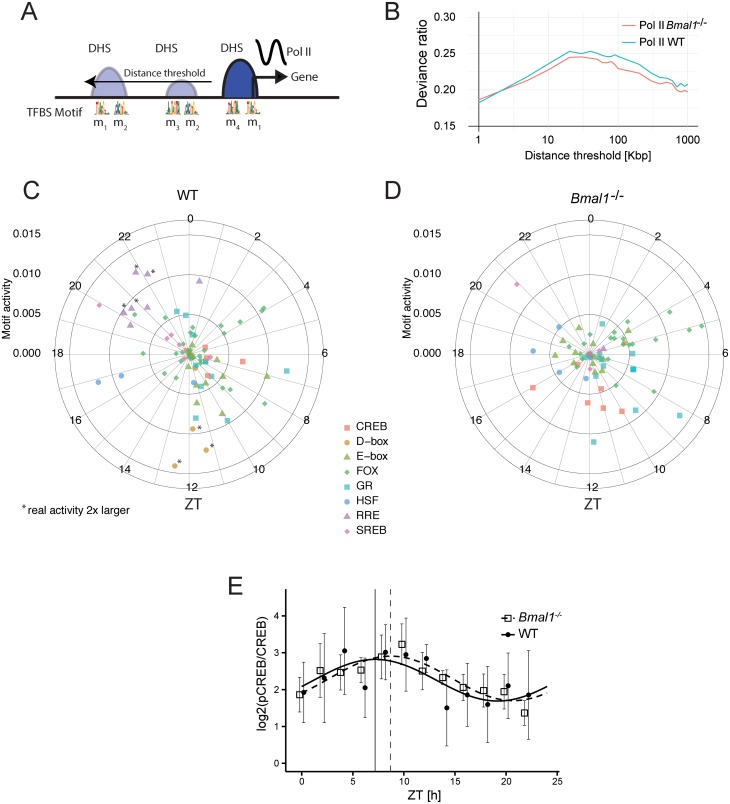
Distal DNase I Hypersensitive Sites (DHSs) help identify diurnally active transcription regulators. A. Scheme of the linear model to infer active transcription regulators: transcription factor (TF) motifs in DHSs within a symmetric window around active transcription start sites (TSSs) are used to explain diurnal rhythms in transcription. B. Fraction of explained temporal variance (deviance ratio) in RNA polymerase II (Pol II) loading (at the TSS of all actives genes) for WT and *Bmal1*^-/-^ mice, in function of the window size (radius) for DHS inclusion, shows a maximum at around 50 kb. Here, *α* = 0 was used in the glmnet (Materials and methods). C–D. Inferred TF motif activities for WT and in *Bmal1*^-/-^ mice shown with amplitudes (distance from center) and peak times (clockwise, ZT0 at the top) using a window size of 50 kb. All 819 (WT) and 629 (*Bmal1*^-/-^) motifs (overlap is 427) with nonzero activities are shown. Note though that most activities are very small and cluster in the center. Certain families of TFs are indicated in colors (full results are provided in S4 Table). Radial scale for activities is arbitrary but comparable in C and D. E. Quantification of western blots for pCREB (Ser 133 phosphorylation) and CREB in WT and *Bmal1*^*-*/-^ genotypes (log_2_ (pCREB/CREB)). Nuclear extracts from four independent livers were harvested every 2 h. Both genotypes showed a significant oscillation (*p* < 0.05, harmonic regression) of the mean signal from the four mice. Though the peak time in *Bmal1*^-/-^ mice is delayed by 1.8 h, the comparison of the rhythm in the two genotypes was not significant (*p* = 0.49, Chow test). Individual blots are shown in S7 Fig.

To identify transcriptional regulators underlying rhythmic transcription by the circadian clock and feeding–fasting cycles, we used a computational approach that combines temporal Pol II loading at TSSs in WT and *Bmal1*^-/-^ mice with annotated TF-binding sites in accessible chromatin regions as defined by our DHSs. Using DHSs and a collection of about 1,900 position-weight matrices for TF–DNA affinities (Materials and methods), we identified DNA sequence motifs that explain rhythmic Pol II patterns in WT and *Bmal1*^-/-^ mice. Briefly, we modified previously described linear models [17,52,53] to identify transcriptional activities (strictly speaking, DNA motifs) represented by phase (time of maximal activity) and amplitude (Materials and methods). In this model, motif activities are linearly combined (as in the phase vector model [54]) according to the presence of corresponding DNA motifs within nearby DHSs. This enabled us to take into account, in addition to the proximal promoter, a collection of putative regulatory regions that may control the expression of a given gene (Fig 4A). Specifically, we considered motifs in DHSs located within a certain window from active promoters, and first estimated the optimal window size according to the quality of the fit. We found that the inclusion of DHSs up to 50 kbp improved the fits in both genotypes (Fig 4B), suggesting that enhancers (represented by distal DHSs) contribute to circadian gene transcription. In WT mice (Fig 4C, S4 Table), our modeling confirmed that known circadian TFs showed the strongest activities, as reflected by the emergence of ROR response elements (RREs) [18,55] with predicted peak activity at ZT22, D-Box elements at ZT12 [56], and E-boxes around ZT8, as previously described [57]. Other motifs that had previously been associated with diurnal transcription in the liver were also identified. These included FOX motifs around ZT20 and ZT5 [58–62], the CREB motifs at ZT7 [63–67], GR motifs around ZT10 [68], SREBP motifs at ZT19 [24,69,70], HSF1 at ZT16 [21,22,26], and ETS TFs in the morning [28]. Further analysis regarding cooccurrences of these motifs at the TSSs overlapping a DHS with cycling DNase I, H3K27ac, and Pol II revealed, among several interesting associations (S6B Fig), that E-box motifs were positively associated with CREB and D-Box motifs but negatively associated with SREBP, FOX, and RRE motifs (S6B Fig). To further substantiate the E-box–CREB association, we selected DHSs overlapping BMAL1 ChIP-seq sites and considered the distribution of CREB motifs as well as the CREB ChIP-seq signal around the BMAL1 peak centers (S6C Fig). CREB motifs were indeed slightly enriched, and a CREB ChIP-seq signal was clearly present on those sites (S6D Fig).

In *Bmal1*^-/-^ mice (Fig 4D, S4 Table), activities of E-box, RRE, and D-Box motifs were not detected or greatly reduced, as expected in the absence of a functional circadian oscillator. On the other hand, TFs linked with metabolic functions, notably those associated with feeding rhythms (e.g., FOX, CREB, SREBP) were identified among the strongest contributors in the absence of a functional clock. Similarly, TFs whose activity depend on systemic signals (e.g., GR and HSF1) were also found with peak activity times that were similar in the WT and *Bmal1*^-/-^ mice. Interestingly, CREB was found among the most delayed TF activities, with a predicted delay of 6 h (S4 Table). To test this prediction, we measured nuclear levels of CREB and pCREB (activated CREB, as measured by phosphorylation on Ser 133) using western blots of nuclear extract from four independent livers every 2 h in WT and *Bmal1*^-/-^ mice (Fig 4E and S7 Fig). This showed an oscillatory pattern of CREB activity in WT mice (as previously reported in [71]), which was still oscillating in the *Bmal1*^-/-^ livers, suggesting that CREB activity is regulated by food-related signaling in clock-impaired mice subjected to a night-restricted feeding regimen. However, while we observed an average phase delay of approximately 2 h in *Bmal1*^-/-^ mice, this trend was not significant (*p* = 0.5, Chow test), presumably owing to interindividual variability in the feeding patterns. Indeed, such interindividual variability is common in metabolism-related, time-dependent regulatory processes, as reported for the rhythmic activation of the TORC1 and AMPK pathways [72].

### BMAL1 has specific impact on DNA accessibility in regulatory regions

**Fig 5:**
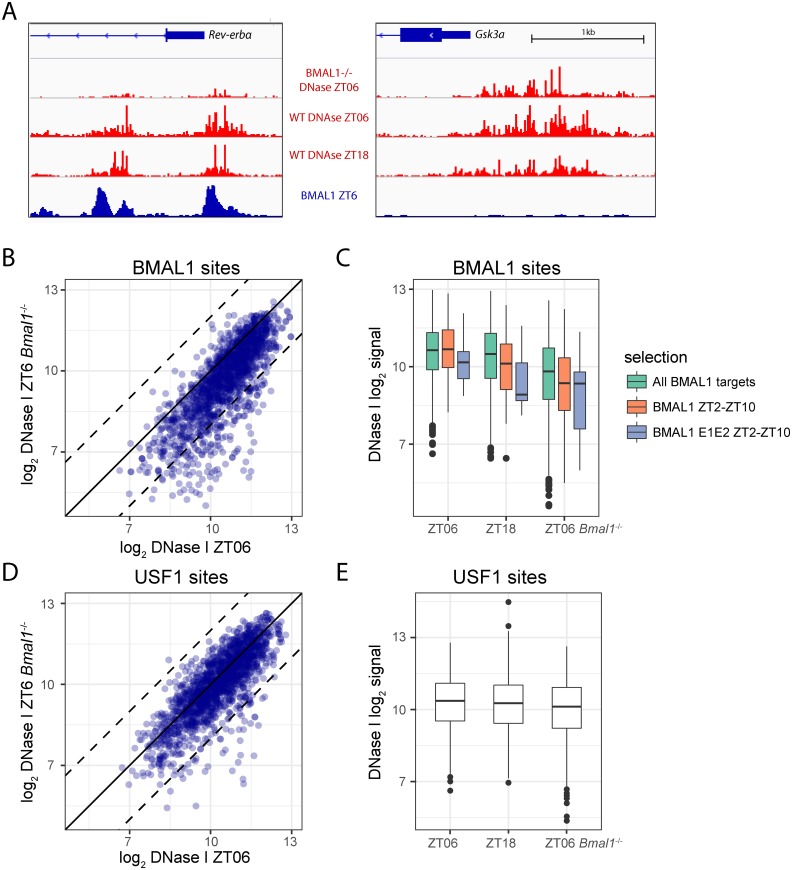
Chromatin accessibility in *Bmal1*^-/-^ mice at ZT6 is generally similar as in the Wild-Type (WT) mice but is lower at BMAL1 sites. A. The *Rev-erbα* (left) and *Gsk3a* (right) promoters. DNase I signal (in red) is strongly reduced in *Bmal1*^-/-^ mice at sites bound by CLOCK:BMAL1 in WT mice (BMAL1 chromatin immunoprecipitation followed by DNA sequencing (ChIP-seq) signal in blue) in the *Rev-erbα* promoter but is similar in WT and *Bmal1*^-/-^ mice at the *Gsk3a* promoter that are not bound by BMAL1. The vertical scale is the same for all three DNase I tracks, as well as for both BMAL1 ChiP-seq tracks. Wild-type ZT18 signals are lower (about half) than at ZT6 in both genes but not as low as in the *Bmal1*^-/-^ mice. B. Comparison of DNase I signals at ZT6 in *Bmal1*^-**/-**^ versus WT mice. All DNase I hypersensitive sites (DHSs) overlapping BMAL1 ChIP-seq peaks in [17] are shown (*n* = 1,555). The dashed lines indicate 4-fold difference. C. Boxplots showing DNase I intensity at the same sites as in B, at peak (ZT6) and trough (ZT18) activities of BMAL1 in the WT, and at ZT6 in *Bmal1*^-/-^ mice for all BMAL1-binding sites (green), BMAL1 sites with an associated expression phase between ZT2 and ZT10 (orange), and with a tandem E-box (grey). All pairwise comparisons (within the same color) between either ZT6 versus ZT18 or ZT6 versus ZT6 *Bmal1*^-/-^ are significant (*p* < 0.001). D–E. Same as B–C but using overlap with USF1 ChIP-seq peaks [74] to select DHSs (*n* = 1,705).

We next examined how BMAL1 binding might influence cycling of activity-related signals (DNase I, H3K27ac, Pol II) at DHSs, as well as DNA accessibility more generally. To address this, we first used our previously published BMAL1 ChIP-seq data [17], which had revealed a highly nonhomogenous peak-strength distribution, showing a handful of very strong peaks associated mostly with circadian and rhythmically transcribed genes (~20) and a majority of weak sites linked to a broad range of genes without obvious rhythmic expression [17]. Consistent with this peak-strength distribution, the percentage of cycling DHSs among BMAL1-overlapped DHSs strongly increased with binding strength (S8 Fig). While we found that DNase I signals oscillate at all the strongest BMAL1 sites (this concerns only about 20 sites), the percentage of BMAL1 sites with cycling DHSs decreased to about 43% for the entire set (S8 Fig). Since tandem E-boxes were found in a large majority of the strongest BMAL1 sites [17], we hypothesized that tandem E-boxes might also promote differential DNase I signals, which we observe below (Fig 5).

Since BMAL1 was proposed to act as a pioneer-like DNA-binding factor [36], we next investigated the effect of *Bmal1* knockout on DNase I signal and thus performed DHS mapping at ZT6 in our *Bmal1*^-/-^ mice [73], near the maximal DNA-binding activity of BMAL1 in WT mice. DNase I hypersensitivity at strong BMAL1 sites (detected in ChIP-seq), such as in the *Rev-erb*α locus, was markedly decreased (by about 5-fold) in *Bmal1*^-/-^ mice, whereas control (unbound) regions like the *Gsk3* promoter showed little difference (Fig 5A). Overall, we observed a clear shift in DNase I hypersensitivity in *Bmal1*^-/-^ mice: namely, regions bound by BMAL1 in the wild type [17] showed, on average, about 2-fold reduced DNase I cuts in *Bmal1*^-/-^ animals (Fig 5B). The effect was markedly stronger when selecting BMAL1 sites more stringently—notably in BMAL1 sites with target gene expression within the ZT2–ZT10 interval—and for such sites that also contained tandem E-box sites (Fig 5C). These findings are consistent with the proposed pioneering function of the BMAL1-CLOCK complex [36]. In comparison, DNase I signals at those sites in the wild-type at the minimum of BMAL1 activity (ZT18) only showed reduction compared to ZT6 for the more stringent selections (Fig 5C), which could reflect residual DNA-binding activity of BMAL1 at the trough time [17]. To verify that the differential DNase I signals were specific to BMAL1 sites, we performed the same analysis at sites bound by the E-box–binding protein USF1 [74], which did not show similar differences between WT and *Bmal1*^-/-^ animals (Fig 5D and 5E).

### DNase I footprints at BMAL1 sites reveal temporal exchanges of transcription factor complexes

**Fig 6:**
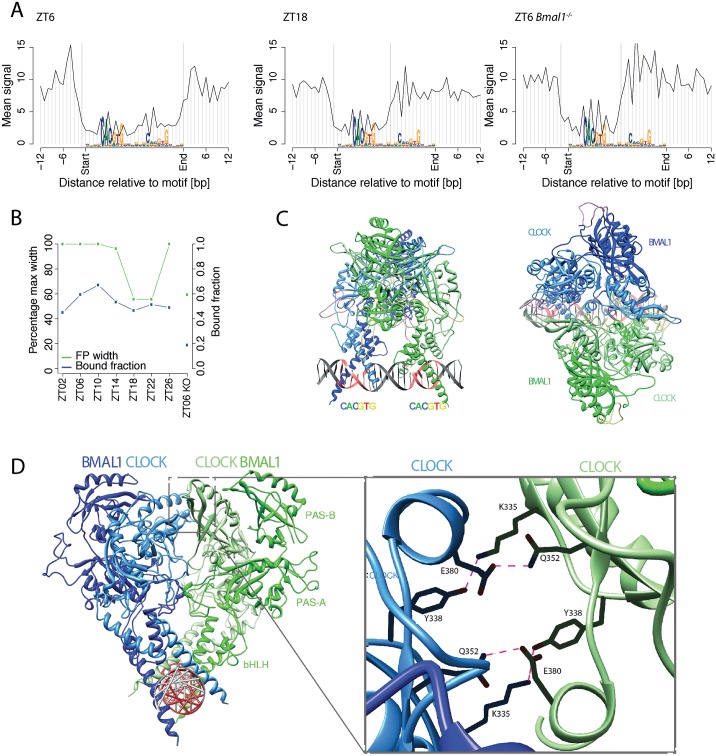
BMAL1 footprints indicate temporally changing protein–DNA complexes, consistent with binding of a heterotetramer to DNA. A. Genomic profiles of DNase I cuts around double E-boxes with a spacer of 6 bp (E1-E2 sp6). We selected *n* = 249 E1-E2 sp6 motifs overlapping a BMAL1 chromatin immunoprecipitation followed by DNA sequencing (ChIP-seq) peak and show the average of profiles for loci classified as bound by the mixture model (posterior probability >0.5). At ZT6, we observed that nucleotides around both E-boxes are protected. In contrast, at ZT18, the width of the protected region is reduced by approximately half, with the second E-box no longer protected from digestion. The signals are anchored to the motif position. Orientation of sites and signals is according to the best match to the E1-E2 sp6 motif. In *Bmal1*^*-/-*^, only one E-box appears occupied. B. Width (left-side *y*-axis, green) of the protected region in WT and in *Bmal1*^*-/-*^ mice for E1-E2 sp6 motifs occupied by BMAL1. Fraction of predicted occupied sites is shown in blue (right-side *y*-axis). C. Two views of the 3-D computational model of the CLOCK:BMAL1 heterotetramer showing two heterodimers of CLOCK:BMAL1 occupying an E1-E2 sp6 site. The two heterodimers are shown in green and blue, while darker green and darker blue correspond to BMAL1 and lighter colors to CLOCK proteins. Information content along the DNA strands is shown in grey with highly constrained nucleotides of the motif in red. D. Zoom on the interacting residuals on the PAS-B domain of CLOCK implicated in the heterotetramer formation.

Owing to the 3-D structures of protein–DNA interactions, genomic patterns of DNase I cleavage around transcription factor binding sites display factor-specific footprints [32,75–78]. We previously showed that BMAL1 binds DNA rhythmically and that strong BMAL1 binding was frequently associated with tandem E-boxes [79] separated by 6 or 7 nucleotides, which were bound by one or two CLOCK:BMAL1 dimers [17]. Here, we analyzed DNAse I footprints at BMAL1-binding sites as a function of time. Starting from BMAL1 ChIP-seq sites, we modified a “mixture model” for DNase I cuts [80] to determine the optimal boundaries of the footprints at each time point as well as the probability that the factor is bound to DNA (calculated here as the probability that the DNase I showed a footprint) for every site (Materials and methods, S1 File). We then analyzed footprints at BMAL1-binding sites containing tandem E-boxes separated by 6 bp (E1E2-sp6). At ZT6, close to the maximal DNA-binding activity of BMAL1, both E-boxes in the E1E2-sp6 motif appeared to be protected from digestion. In contrast, at ZT18, only the 5′ E-box displayed a footprint consistent with occupation by a transcription factor (Fig 6A, full time course in S9 Fig). Moreover, the footprint at ZT18 was undistinguishable from that in the *Bmal1*^-/-^ mice, suggesting that other transcription factors bind BMAL1 sites when BMAL1 activity is low. The estimated proportion of E1E2-sp6 motifs showing a footprint indicative of two CLOCK:BMAL1 dimers varied across time points, with a maximum of 65% at ZT10 and minimum of 20% in the *Bmal1*^-/-^ animals (Fig 6B). Also, the binding dynamics of BMAL1 at E1-E2-sp7 (tandem E-boxes separated by 7 bp) was largely similar to that for E1-E2-sp6, though E1-E2-sp7 had both E-boxes predominantly protected only at ZT6, suggesting spacer-specific binding dynamics (S10 Fig). In contrast, the footprints at BMAL1-binding sites with single E-boxes did not show significant changes in time or in the *Bmal1*^*-/-*^ mice (S11 Fig), again suggesting that other bHLH transcription factors can also bind at BMAL1 sites. In fact, footprints at DNA regions bound by the bHLH transcription factor USF1 in ChIP-seq [74] were largely similar to that of BMAL1 sites with single E-boxes, though the fraction of sites with clear footprints was reduced for USF1 compared to BMAL1 (S12 Fig).

To better understand the time-dependent footprint at BMAL1 sites and to gain insight into how the CLOCK:BMAL1 heterodimer occupies its tandem E-box–containing target sites, we used recently established 3-D protein structures of single CLOCK:BMAL1 complexes combined with molecular modeling (Materials and methods). Our models strongly support formation of CLOCK:BMAL1 heterodimers in a heterotetramer configuration at peak activity of these factors and residual binding of the dimer or other transcription factors during low-activity times. Two 3-D models of the heterotetramer configuration were constructed. In the first model, the spacing between the two E-boxes was 6 bp (sp6) (Fig 6C, S3 Movie, S9 Fig, S2 File), and in the second model the spacing was 7 bp (sp7) (S4 Movie, S10 Fig). For the model of the single CLOCK:BMAL1 complex, we used the crystal structure of the heterodimeric CLOCK:BMAL1 (Protein Data Bank [PDB] id: 4F3L) [81], into which we built the missing parts of the flexible loops. To link the single CLOCK:BMAL1 model to the E-box, we employed the complex crystal structure of CLOCK:BMAL1 basic helix–loop–helix domains bonded on the E-box (CACGTG) (PDB id: 4H10) [82]. We then superimposed the two single CLOCK:BMAL1 E-box models with the sp6 DNA and the sp7 DNA, forming the respective symmetric heterotetramer models. We found that the 6-bp spacing between the two E-boxes was optimal to establish favorable interactions between the two CLOCK:BMAL1 heterodimers (Fig 6D), involving mainly residues (e.g., K335, Y338, Q352, E380, and E384) located in the PAS-B domain of the CLOCK in a dynamic H-bond network [83]. Similarly, the 7-bp spacing seemed also able to favor a heterotetramer conformation, producing only a minor twist of 10° in the three interval base pairs. However, a conformation with base-pair spacing less than 6 or more than 7 would make complex formation difficult because of conformational constraints. Thus, the modeling results are consistent with two CLOCK:BMAL1 heterodimers binding to two E-boxes separated by 6 or 7 base pairs, and the DNase I footprints with characteristic and dynamically changing shapes suggest exchanges of different transcription factor complexes on the DNA during the diurnal cycle.

Finally, we examined temporal footprints at DHSs bound by other rhythmically active TFs: REV-ERB, HSF1, SREBP, and CREB (S13 Fig). Interestingly, and unlike what we observed for CLOCK:BMAL1, the shapes of the footprints for those factors did not change with time and were unaffected in the absence of BMAL1. However, the fraction of sites showing footprints coincided well with the maximal transcriptional activity of the different factors. For example, footprints centered on REV-ERBα–bound RREs showed the largest proportion of footprints at ZT22, which coincides with the trough activity of the REV-ERB repressors. The low percentage of bound (as detected in ChIP) RREs with footprints called by the model was low (<20%), which could reflect that nuclear receptors tend to have a low residence time and therefore display a lower DNase I cleavage-protection pattern [77]. For HSF1, the number of footprints was maximal at ZT18—approximately 4 h later than the previously reported peak activity [84]—and for the feeding-induced SREBP this number peaked during the night, as expected [24]. Lastly, high-confidence CREB-binding sites [85] showed clearly marked and invariable-width footprints throughout the 24 h in both WT and *Bmal1*^-/-^ mice, consistent with the finding that CREB activity is regulated posttranslationally on the DNA [63–66].

## Discussion

### DNase I hypersensitivity shows daily rhythms in the adult mouse liver in sync with transcription and chromatin activity marks

We mapped genome-wide DNase I hypersensitivity with 4-h time resolution in adult mouse livers. While most of the signal is likely derived from hepatocytes that constitute 80% of the liver mass, we cannot exclude that some signals, including rhythmic ones, originate from the remaining nonparenchymal cell types. Our analysis provided a comprehensive view on the dynamics of chromatin accessibility controlled by the circadian clock and feeding–fasting cycles. Overall, the identified hypersensitive regions clustered in about 60,000 DHSs. The latter tended to span several hundreds of base pairs and collectively covered 2% of the mappable genome. One-third of these regions were located near gene promoters and the remaining two-thirds were more distal from TSSs, which is consistent with what has been previously observed in mammalian cells [40]. On a genome-wide scale, 98,000 footprints were detected in about 60% of these accessible regions. Importantly, our data provided global insights into the temporal variations in DNase I hypersensitivity on the timescale of several hours to a day. Indeed, while it was previously shown that high-amplitude circadian genes such as *Dbp* showed nearby hypersensitive regions [30], it was not known how widespread these rhythms are across the genome. Here, we showed that thousands of DHSs exhibit rhythmic signals with peak-to-trough amplitudes that are comparable to those of Pol II signals. Accessible chromatin, as measured with DNase I hypersensitivity, is typically associated with transcriptionally active states and often reflects the presence of proteins bound to regulatory DNA elements [31,32,40]. However, we showed that DNase I–sensitive regions within gene bodies, notably in the case of highly transcribed genes, may reflect transcription elongation. As a consequence, they do not necessarily display DNA footprints such as the ones discerned in regulatory elements.

We then compared the temporal patterns of DNase I hypersensitivity with other frequently used transcriptional activity marks, in particular H3K27ac and Pol II. While DNase I signals, H3K27ac, and Pol II densities all showed abundant rhythmicity, H3K27ac abundance cycled at the largest number of DHSs, in particular at distally located sites. For both DNase I hypersensitivity and H3K27ac, the peak-to-trough amplitudes appeared higher in distal elements as compared to TSSs. Such dynamic accessibility might reflect increased protein-binding dynamics at enhancers, suggesting their potential role in controlling diurnal gene expression. This would parallel mechanisms underlying cell-type specificity, in which the modulation of histone marks and accessibility of chromatin at enhancers are among the major features associated with regulatory mechanisms [86]. The hypothesis that distal DHSs might represent enhancers for diurnal transcription was further supported by our observation that rhythms in pairs of putative enhancers and nearby TSSs showed a tight temporal correlation. In contrast to the observed delay between H3K4me3 enrichment and Pol II density, reported previously [12], no significant delays were observed between accessibility as measured by DNase I hypersensitivity and H3K27ac enrichment. This likely reflects that turnover of histone acetylation is faster than that of histone methylation [87]. We then used these temporal datasets to explore the involvement of putative enhancer regions in the cyclic recruitment of Pol II at the TSSs and subsequent transcription of the respective target genes. Our findings were consistent with a previous study on eRNA, which showed that eRNAs cluster in specific circadian phases and are correlated with Pol II occupancy and histone acetylation [6,28]. In addition, eRNA levels are correlated with the expression of nearby genes [28].

### BMAL1 knockout animals subjected to a nighttime feeding regimen show widespread Pol II and mRNA rhythms

Our genome-wide study of Pol II loading and mRNA expression in WT and *Bmal1*^-/-^ mice kept under LD cycles and night-restricted feeding revealed that the number of genes exhibiting diurnal fluctuations did not drastically change in these behaviorally arrhythmic animals. This was a rather unexpected finding; however, we also found that the number of high-amplitude oscillations in mRNA accumulation was much reduced in *Bmal1*^-/-^ mice. In addition, we observed greater differences for mRNAs than for Pol II loading. Given our previous research [12] demonstrating little, if any, significant regulation of circadian gene transcription at the elongation level, these differences suggest the involvement of posttranscriptional mechanisms in controlling circadian gene expression, which is well documented [11,13,14]. All in all, our observations suggest that feeding cycles can entrain a significant set of low-amplitude transcriptional oscillations, while the circadian clock drives high-amplitude rhythms of a relatively limited number of transcripts (S4 Fig).

### Combining DHSs with genomic sequence can predict transcription factors with cycling activities in the presence or absence of BMAL1

In this study, we accumulated compelling evidence for the contribution of distal regulatory elements in circadian transcription regulation. In fact, we observed that about 47% of DHS are located at more than 10 kb from the closest active TSS. Using penalized regression models, we predicted a collection of transcription factor binding motifs that best explain diurnal variation in transcriptional activity in both WT and *Bmal1*^*-/-*^ mice. Moreover, while the analysis of promoter sequences recently yielded insights into promoter architecture that favor rhythmic transcription [88], the inclusion of distal DHSs up to 50 kb improved the variance explained by our penalized linear model in WT and *Bmal1*^*-/-*^. The obtained set of transcription factors that exhibited high-activity amplitudes in WT mice was similar to the one derived from a screen that used differential display of DNA-binding proteins [21]. On the other hand, comparison with *Bmal1*^-/-^ mice indicated that transcription regulators related to feeding–fasting cycles and rhythmic systemic signals were active in both genotypes, as would be expected. Among those, forkhead domain factors (FOX) have been implicated in cell cycle regulation and oxidative stress and are negatively regulated by insulin signaling [59]. Notably, FOXO1 and FOXO6, like the core clock [4], regulate the expression of key enzymes implicated in gluconeogenesis [60,89], collectively pointing towards FOX transcription factors as effectors of metabolic rhythms in liver.

We also found CREB to be among the most delayed transcription factor activities inferred by the generalized linear model in a *Bmal1*^*-/-*^ mouse liver. CREB is implicated in the nutrient response cycle and it regulates hepatic gluconeogenesis [62,63,68,71]. We were able to replicate the pattern of CREB activity in WT mice [71] and we showed that CREB activity is still oscillating in *Bmal1*^*-/-*^ mice, indicating that CREB is regulated by food-related signaling. Consistently, CREB activity during fasting was shown to be modulated by CRY1 and CRY2, which are rhythmically expressed in the liver [71]. Similarly, TFs that are responsive to systemic signals—such as HSF1 driving rhythmic transcription of heat shock proteins around ZT18 [21,22] or the GR sensitive to glucocorticoid hormones (GCs) released near the day–night transition [25,90–93]—were identified both in WT and *Bmal1*^-/-^ mice. Our identification of GR activity is consistent with the previous observation that hundreds of circadian transcripts, distinct from clock-controlled circadian genes, are under glucocorticoid control [94]. In summary, our analyses revealed an important number of genes that in the absence of the clock have higher cycling amplitude or even cycle de novo. Many of these genes have a link with metabolism, which could indicate a role for the clock in buffering nutrient-induced rhythmicity of metabolic gene expression.

CLOCK:BMAL1 complexes recruit coactivators (p300 and CBP) or corepressors (CRYs, PERs). At tandem E-box sites, these ChIP signals for BMAL1, CLOCK, and NPAS2 from [11,17] were clearly more enriched at CT8 compared to CT20 and higher at tandem compared to single E-boxes (S14 Fig). On the other hand, corepressors (CRYs and PERs) were clearly more abundant on DNA at CT20 (the effect for CRY1 was weaker but this was discussed already in [11]). Moreover, at CT20, the repressors showed a weaker signal at tandem E-boxes compared to single E-boxes. Finally, the cofactors p300 and CBP exhibited an opposite behavior: while p300 behaves similarly to BMAL1, CBP behaved like PER or CRY, suggesting a differential regulatory function.

### Transcription factor binding dynamically reshapes DNA footprints

Comparing DNase I signals between WT and *Bmal1*^*-/-*^ samples at ZT6 revealed that BMAL1-binding sites showed on average a decrease in DHS signals (Fig 5), which may be consistent with a pioneering function for the CLOCK:BMAL1 core clock transcription factor [36]. Moreover, our analysis of DNase I signals at nucleotide resolution revealed interesting dynamics in the shape of the footprint, which was reminiscent of our earlier proposition that strong and functional CLOCK:BMAL1 recognition elements—as with those found near the majority of core circadian clock genes—involved the binding of a dimer of CLOCK:BMAL1 heterodimers [17,74,79,83]. Here, we found that CLOCK:BMAL1 binding leaves a wide footprint spanning a tandem E-box element at the maximum activity and that this footprint shrinks to encompass only a single E-box at the minimum activity time point, resembling that footprint detected in *Bmal1*^-/-^ mice. This indicates that other E-box–binding transcription factors expressed in the liver, such as USF1, can occupy these E-box sites. These transcription factors may thereby function as placeholders to render these sites quickly accessible for CLOCK:BMAL1 heterodimers at the onset of the next circadian cycle. Indeed, USF1 has been shown to act as a nonallelic suppressor in certain mouse strains carrying a semidominant mutation of CLOCK [74]. Structural modeling of the TF–DNA complexes based on the CLOCK:BMAL1 crystal structures supported the establishment of a heterotetramer configuration at peak activity [17,74,79,83]. Other transcription factor footprints, (S13 Fig) such as for HSF, SREBP, RRE, and CREB, did not show a changing footprint shape. In the case of RRE, this might reflect a dynamic exchange of activating and repressing TFs (namely RORs and REVERBs) on their cognate binding sites [95].

## Conclusion

We performed temporally resolved DNase I hypersensitivity mapping to identify regulatory elements and transcription factor footprints underlying rhythmic transcription during diurnal cycles in the mouse liver. Our study sheds light on the interrelationships between the nutrient response cycle and the circadian clock as well as the contribution of the distal regulatory elements to circadian gene expression. In sum, we found that hypersensitivity at both promoter proximal and distal sites oscillates in phase with transcription during diurnal cycles. Computational integration of DHSs with transcription activity allowed us to highlight differences in the transcriptional regulatory logic of diurnal cycles in WT and circadian clock–deficient *Bmal1*^*-/-*^ animals. Finally, digital footprint analysis revealed dynamically changing transcription factor complexes on DNA.

## Materials and methods

### Ethics statement

All animal care and handling was performed according to the Canton of Geneva (Ueli Schibler, authorization no. 1010/3950/0) and Canton of Vaud (Nouria Hernandez, authorization no. VD2546.2 and Fred Gachon, authorization no VD 2720) laws for animal protection.

### Animals

C57/BL6 male and *Bmal1*^*-/-*^ mice [73] 12–14-wk-old (at time of euthanasia) were housed in a 12-h light/12-h dark (LD) regimen. Starting 7 d before euthanasia, the mice were entrained to a 12 h/12 h LD regimen with ad libitum access to water. For the Pol II ChIP-seq, H3K27ac ChIP-seq, and microarray experiments, food was accessible only between ZT12 and ZT24 (restricted feeding). Mice used for DNase I-seq were entrained to a 12 h/12 h LD regimen with ad libitum access to both water and food. At each ZT2, ZT06, ZT10, ZT14, ZT18, ZT22, and ZT26, three to five mice were anesthetized with isoflurane and decapitated. The livers were perfused with 2 ml of PBS through the spleen and immediately collected. A small piece of liver tissue (approx. 100 mg) was snap-frozen in liquid nitrogen and kept at −80°C for RNA extraction. The remaining liver tissue was immediately homogenized in PBS containing 1% formaldehyde for chromatin preparation.

### DNase I-seq

Mouse liver nuclei were prepared as described in [96]. Freshly prepared nuclei were suspended in ice-cold Ψ-buffer (11 mM KPO_4_ at pH 7.4, 108 mM KCl, 22 mM NaCl, 5mM MgCl, 1 mM CaCl_2_, 1 mM DTT) and pelleted. 5 x 10^6^ nuclei were suspended in 200 μl of Ψ-buffer supplemented with 0.2% of NP40 and 1 u/ml of DNase I (DPFF Worthington Biochemical Corporation). DNase I digestion was performed for 6 min at room temperature and the reaction was stopped by adding 200 μl of lysis buffer (50mM Tris-HCl at pH 8, 20 mM EDTA, 1% SDS, 200 μg/ml proteinase K). Protease digestion was performed overnight at 55°C. RNaseA (100 μg/ml) was then added and samples were incubated at 37°C for 1 h. DNA was then extracted twice with phenol-chloroform and precipitated with isopropanol in the presence of 0.5 M NaCl. DNAs were dissolved in 5 mM Tris-HCl pH 8. DNA from four animals were pooled, and 75 μg of DNA was loaded on 11 ml 10%–50% sucrose gradient in STE buffer (1M NaCl, 20 mM Tris-HCl at pH 8, 5 mM EDTA) and centrifuged at 30,000 rpm for 16 h at 20°C (SW 40 Ti rotor, Beckman Coulter Inc). The sucrose gradients were then fractionated, and DNA was precipitated by two volumes of ethanol in the presence of 5 μg of glycogen. Fractions containing DNA sized around 300 bp were pooled and used for Illumina library preparation.

### ChIP-seq of RNA polymerase II

For *Bmal1*^*-/-*^ animals, perfused livers were processed for chromatin preparation as described in [16]. The chromatin samples from the five mice were then pooled, frozen in liquid nitrogen, and stored at –80°C. For the ChIP experiments, the following antibodies were used: anti-RPB2 (Santa Cruz Biotechnology, sc-673-18). To determine the optimal amounts of each antibody, we performed pilot ChIP assays and determined the enrichment for a set of promoters by real-time qPCR according to [16]. A total of 1 ml of each chromatin suspension (containing about 60 μg of DNA) was incubated with 10 μg of anti-RPB2 in buffer A (20 mM Tris/HCl [pH 7.5], 150 mM NaCl, 2 mM EDTA) overnight at 4°C on a rotating wheel. 10 μl of protein A bead suspension (25% slurry in buffer A), preblocked with 10 μg/ml of salmon sperm DNA and BSA at 4°C overnight, was then added and the incubation was continued for 1 h at room temperature on a rotating wheel. The beads were then washed with dialysis buffer and ChIP wash buffer as described in [97]. Protein–DNA complexes were eluted from the beads, de-crosslinked, and treated with RNase A (and subsequently with proteinase K) as described in [16]. The DNA concentration was determined by fluorometry on the Qubit system (Invitrogen). A total of 10–12 ng DNA was used for the preparation of the library. Libraries for ultra-high-throughput sequencing were prepared with the ChIP-Seq DNA sample kit (Illumina) as recommended by the manufacturer.

### ChIP-seq H3K27ac

For WT and *Bmal1*^*-/-*^ animals, H3K27ac ChIPs were performed according to the method described by [98] with a few modifications. The 100-μl chromatin aliquots were used for each IP and diluted with 900 μl of RIPA buffer (1% NP-40, 0.5% sodium deoxycholate, 0.1% SDS in PBS at pH 7.4) and added to Dynal magnetic beads conjugated with sheep antimouse IgG dynabeads (Invitrogen, cat no: 110–31) pretreated with 3 μl of polyclonal antibody for H3K27ac (active motif, cat no: 39135) for immunoprecipitation of specific complexes. The samples were incubated overnight at 4°C on a rotator, then magnetic beads were washed seven times with lithium chloride wash buffer (100 mM Tris at pH 7.5, 500 mM LiCl, 1% NP-40, and 1% sodiumdeoxycholate) and once with 1X TE buffer (10mM Tris-HCl at pH 7.5, 0.1 mM Na_2_EDTA). The chromatin complex was eluted using elution buffer (1% SDS, 0.1 M NaHCO_3_) for 1 h at 65°C using Eppendorf thermomixer. The chromatin was then de-crosslinked overnight at 65°C and ChIP DNA purified using Qiagen PCR purification kit and eluted in 50 μl of elution buffer. For qPCR reaction, 1.5 μl of 1/10 diluted ChIP DNA was used. Libraries for ultra-high-throughput sequencing were prepared with the ChIP-Seq DNA sample kit (Illumina) as recommended by the manufacturer.

### ChIP-seq of HSF1

ChIP-seq of HSF1 was performed according to the method described by [17]. The HSF1 polyclonal antibody was from Stressgen (Enzo Life Sciences, ADI-SPA-901). For each IP, 5 μl of HSF1 antibody was used with 250 μl of precleared chromatin. A ChIP library was prepared using four independent ChIP experiments at ZT14, and one lane was sequenced to obtain about 20 million uniquely mapped reads.

### CREB and pCREB western blot on nuclear extract

Hepatic nuclear proteins were prepared as described in [27] using the NaCl-Urea-NP40 (NUN) procedure. 10 μg of the nuclear protein extracts was fractionated on an SDS-PAGE and transferred to a PVDF membrane for western blot analysis. Antibodies against CREB (Chemicon # AB3006) and phospho-CREB (pSer133) (Chemicon #AB3442) were used at 1:1,000 dilutions. Membranes were stained with naphtol blue black in order to quantify the protein loading.

### ChIP-seq and DNase I-seq data analysis

At each time point, DNA sequenced reads were mapped to the mouse genome (*Mus musculus* NCBIM37 genome assembly [mm9; July 2007]) using bowtie through the HTS station portal (http://htsstation.epfl.ch) [99]. Duplicate reads were kept to avoid saturation because of high coverage of Hi-seq libraries and DNase I specificities. Quality controls, including the percentage of reads within enriched regions, indicated high overall enrichment at all time points, as about 50% of DNase-seq reads mapped to 1.3% of the genome considered to be accessible (S1 Table). Peak calling was done using ChIP-peak [100] (http://ccg.vital-it.ch/chipseq/chip_peak.php) on DNase I signals merged from all ZT time points with the parameters: cutoff = 100, vicinity = 400, window size = 600, threshold = 1,000. After peak calling, DNase I, Pol II, and H3K27ac signals were quantified at each time point within a window of ±300 bp around every peak center (±1 kb for H3K27ac). The values thus obtained were quantile normalized between time points for each mark. These quantile normalizations were then applied (by interpolation) to the genomic profiles in Figs 1 and 5. The wig files (S1 Fig) provided in GEO are normalized only to the library sizes.

### Detection of active transcripts

Using ChIP-seq data for Pol II, H3K4me3, H3K36me3, and H3K27ac from [12] in the WT condition, a support vector machine classifier (SVM) was used to detect active transcripts among all Ensembl-annotated transcripts (version NCBIM37). We selected regions of interest to be ±300 bp around the TSS for Pol II and H3k4me3 and also ±300 bp around the TES for DNase I, and the last 600 bp of each transcript for the gene body mark H3K36me3. Read counts on the same strand as the transcript annotation were counted per 10 bp and quantile normalized across time. For training, a set of active and inactive transcripts were extracted, consisting of the top 10% and bottom 10% respectively, as determined by Pol II RPKM along each transcript. An SVM was trained on these active versus inactive transcripts and subsequently applied to all transcripts at each time point. Cross-validation indicated that the SVM had satisfactory false positive and false negative results for very high or very low Pol II signals (98% of test transcripts were correctly classified as either active or inactive at ZT10). Transcripts shorter than 600 bp were set to “active” if they had higher Pol II RPKM than the lower quartile of active transcripts. Transcripts were considered active when they were classified as active at minimally one time point. The active transcripts list was used to associate DHS with the closest active TSS. The annotation result provided 13,457 unique active genes linked with at least one DHS.

### Rhythmicity analysis and selections of DHSs

Rhythmicity analysis was done as previously described [12] using harmonic regression. Throughout (DHS signals, ChIP signals, mRNA expression), log_2_ normalized signals were used in the harmonic regressions. The Fisher combined probability test [50] for Pol II, H3K27ac, and DNase I signals was computed to select rhythmic DHSs. This uses a chi-squared distribution with 2k (k = 3 marks) degrees of freedom. The resulting *p*-value was used to estimate FDRs via the linear step-up method. mRNA microarray in WT and in *Bmal1*^-/-^ mice from [12] were reanalyzed using harmonic regression.

### Analysis of published ChIP-seq data

Published datasets of ChIP-seq of CREB [63], USF1 [74], and REV-ERBα [101] in the mouse liver were quantified in our DHSs. ChIP-seq experiments such as SREBP [24] and BMAL1 [17] were included. Z-scores were computed for each ChIP-seq in each DHS. Z-scores greater than 2 were used for subsequent footprint analysis in Fig 6.

### Footprint detection in DHSs

Footprints in DHSs were detected using Wellington (pyDNase library) [46] with parameters: -sh 20,36,5 -fdr 0.05 on all DNase samples concatenated. To analyze the shape of footprints, we extended a mixture model for DNase I cuts [80] to determine the optimal boundaries of the footprints at each time point as well as the probability that the factor is bound to DNA (calculated here as the probability that the DNase I showed a footprint) for every site (details in S1 File).

### Linear model for inference of phase specific motif activities

To identify rhythmic TF activities from temporal Pol II data, we adapted existing methods based on linear regression [102] to the circadian context [54]. Specifically, we estimated transcription factor motif activities *A*_*f*_ by fitting the following linear model:

$$P_{g}=\Sigma_{f}N_{gf}A_{f},$$

where *P*_*g*_ denotes the 24-h component of the temporal Pol II profile (P_t_) for gene *g* i.e., *P*_*g*_ = Σ_*t*_*P*_*t*_*e*^*iωt*^, with $\omega =\frac{2\pi }{24}h^{-1}$. In practice, to perform linear regression with real numbers, we used real and imaginary parts as two-dimensional vectors. The matrix *N*_*gf*_ represents the susceptibility of gene *g* to the factor *f* and contains the motif content for factor *f* in all DHSs within a certain window of an active TSS (Fig 4A). To cover a large representation of TF motifs, we used FIMO [103] and scanned our DHSs using sets of position weight matrices (PWM) from JASPAR [104], TRANSFAC [105], SELEX [106], and WANG [107] (in total, ~1,900 matrices). We counted all motifs below a threshold of 10^−4^. The fitting was performed using the Elastic-net penalized linear regression model [102], which conveniently controls sparseness of the solution (in virtue of the L1 norm), grouping of redundant features (owing to the L2 term, this was important here, since we have a large and redundant set of matrices), and overfitting (using cross-validation). This method is available as an R package called GLMNET and uses the elastic-net penalized regression. Unless otherwise stated, we used an “alpha” (tunes the relative weights of the L1 and L2 penalties) value of 0.1. In Fig 4C and 4D, real and imaginary parts of the inferred activities *A*_*f*_ are plotted, showing both their amplitudes and peak activity times (phases).

### 3-D structures of CLOCK:BMAL1 heterotetramer

For the single CLOCK:BMAL1, the crystal structure of the heterodimeric CLOCK:BMAL1 (PDB id: 4F3L) was used as an initial model [81]. In this structure, there are five flexible loops lacking density. The residues in positions 129–134 (length of 6 residues), 212–237 (26 residues), 257–275 (19 residues), and 291–309 (19 residues) were missing from BMAL1, and the residues 224–247 (24 residues) were missing from CLOCK. These missing parts were computed by Rosetta's loop modeling application (v3.5), an application that extensively remodels the backbone of the loops [108]. The loops were remodeled and refined by the CCD (Cyclic Coordinate Descent) algorithm [109]. The fragment files used by CCD were made by Robetta Server [110]. The CLOCK:BMAL1 structure, as a unique chain, was used as Rosetta input, and from the output we selected the lowest-energy loops for the single CLOCK:BMAL1 model. In order to bind the single CLOCK:BMAL1 model to the E-box, the complex crystal structure of CLOCK:BMAL1 basic helix–loop–helix domains bonded on the E-box (CACGTG) (PDB id: 4H10) was used [82]. This structure was superimposed to the single CLOCK:BMAL1 model with the UCSF Chimera visualization program (v1.5.3) [111]. In accordance with this superposition, the N-terminal helices of CLOCK and BMAL1 were replaced by the helices in the 4H10 structure from the protein data bank. The base-pair geometry of the DNA in the 4H10 structure was analyzed by the 3DNA software (v2.0) [112]. Two double-strand DNA models, spacing 6 (sp6) and spacing 7 (sp7), with sequence 5′-CACGTGAAAAAA(A)CACGTG-3′, were generated by 3DNA. The CACGTG parts were rebuilt based on the analysis of the DNA in the 4H10 structure. The spacer of 6 bp was built with the standard B-DNA backbone conformation for A–T pairs. For the final models, two CLOCK:BMAL1:E-box models were bound to the DNA models with a spacer of 6 bp (sp6) or 7 bp (sp7) by superimposing them with UCSF Chimera. In the sp6 model, we performed energy minimization for 12,500 steps with the NAMD simulation package v2.9. The model was parameterized by the AMBER force field (ff99bsc0) [113].

### Data visualization

Wig files were generated using the bam2wig script [99] and were normalized by the number of mapped reads divided by 10^7^. DNase I signal is represented using the first position of the read alignments considered as the cutting position and without shifting strands. Pol II and H3K27ac are represented using the coverage by the whole read length after shifting forward (in the read orientation) by 80 bp and 90 bp for Pol II and H3K27ac, respectively. These wig files were then visualized on the UCSC genome browser (http://genome.ucsc.edu/).

## Supporting information

S1 Fig DNase I accessibility around *Dbp* locus. Measured DNase I-seq signals near the *Dbp* gene, compared with previously reported DHSs in a reference study [30] (marked site_1 to site_7). [30] found seven hypersensitive sites while we detected six DHSs using our peak calling at compatible locations (black marks). Moreover, [30] reported high (sites 2, 4, 6, and 7, in green), or lower (sites 1, 3 and 5 in blue), amplitudes in rhythmic DNase I digestion efficiency, consistent with the DNase I-seq signals (visual inspection). Sites 2, 4, and 7 contain E-boxes that are binding sites for CLOCK and BMAL1. Locations of BMAL1 ChIP-seq signals (bottom track) [17] clearly overlaps strongest DNase I peaks. DNase I browser tracks are normalized to the total read count here.
(PDF)

S2 Fig Genomic characteristics of DHSs. A. Distribution of distances between DHSs and nearest active TSSs. We observe a bimodal distribution, with a first mode corresponding to DHSs in promoter regions (centered on 100 bp from the TSS) and a second mode centered on 10 kb from TSSs. B. Repartition of DHSs within three classes depending on their distance from the nearest TSS: 47% are more than 10 kb from a TSS and are classified as distal, 28% are between 1 kb and 10 kb away and are classified as proximal, and DHSs located 1 kb or less from a TSS represent 24% of all sites. C-D. Pol II, DHS and H3K27ac signals around TSSs and distal DHSs (averages over all sites). Profiles were normalized so that the maximum around the TSS is 100%. E. DNase I signals (all time points are merged in the ZT All track) near the *Albumin* gene. Footprint detected using the Wellington algorithm are shown below the detected DHS sites. The promoter region is enlarged at the bottom, showing that the wide footprint detected in our data corresponds to previously established transcription factor binding sites (the colored boxed indicate protein complexes previously identified in [47]). Many sensitive regions locate din the gene body do not display footprints, probably due to high transcription of *Alb* in the liver.
(PDF)

S3 Fig Phase relationships between DHS, Pol II, and H3K27ac at all DHSs outside transcribed regions. Similarly to Fig 3D, high correlations and no phase shifts can still be observed outside of actively transcribed regions, demonstrating that this relationship is not only linked to active transcription.
(PDF)

S4 Fig Diurnal oscillations in transcription and mRNA accumulation in WT and *Bmal1*^-/-^ livers. A. Number of oscillating genes in WT and in *Bmal1*^-/-^ mice using Pol II loadings at TSSs and mRNA. B. Cumulative count of oscillating genes (selected with p < 0.05, harmonic regression) in *Bmal1*^-/-^ and WT mice with log_2_ amplitude greater or equal than the values on the x-axis. Both Pol II loadings at TSSs and mRNA are shown. Values below 0.5 on the x-axis are not shown. C. Peak times (ZT times) of genes oscillating in WT and in *Bmal1*^-/-^ using Pol II loadings at TSS. D. Idem using mRNA accumulation profiles.
(PDF)

S5 Fig Functional enrichment analysis of mRNAs cycling in wild-type and *Bmal1*^-/-^ animals. A. Boxplot of log_2_ mRNA amplitude of genes from significantly enriched Kegg or Reactome pathway in WT and in *Bmal1*^-/-^ genotypes. These pathways were retrieved using g:Profiler with p<0.1 in one or both genotype. Boxplots were generated with the amplitude of each oscillating genes annotated with a specific pathway. Genes used for each annotation are reported in S3 Table. B. Phase and amplitude of mRNAs for significantly enriched annotations in WT and in *Bmal1*^-/-^ such as circadian rhythm, cholesterol biosynthesis, bile secretion, triglyceride biosynthesis, ribosome biogenesis and M/G1 transition associated genes, reported in a polar scatter plot. Green arrows represent mRNA with higher amplitudes in *Bmal1*^-/-^ mice, while red arrows represent genes that loose rhythmicity in the knockout.
(PDF)

S6 Fig mRNA accumulation in WT and *Bmal1*^-/-^ livers, stratified by ChIP-seq signals and motifs for clock and nutrient transcription factors. A. Cumulative count of genes with oscillating mRNA accumulation (selected with p < 0.05, harmonic regression) in *Bmal1*^-/-^ and WT mice, with log_2_ amplitude greater than the values on the x-axis. The different panels show genes bound by core clock (CLOCK, BMAL1), nutrient-related (CREB and SREBP1) and systemic signal (HSF1) TFs. B. Co-occurrence of DNA motifs on TSSs with cycling DNase I, H3K27ac and Pol II (Fisher’s combined p-value below 0.05). Combinations between DNA motifs for the core clock (E-box, D-box, RRE), the feeding-fasting cycle (FOX, CREB, SREB) and response to systemic cues (GR, HSF) are shown (hypergeometric test, p<0.05). Positive associations are shown in blue and negative associations in orange. C. Motif analysis around BMAL1 ChIP-seq sites [17] for CREB, E-Box and RRE weight matrices (matches use p < 0.001, Oprof in [23]). This motif analysis was performed with a sliding window of 60bp and a 10 bp shift using Oprof from the Signal Search Analysis server. D. ChIP-seq signal for CREB (Fasted conditions, from [63] around BMAL1 sites, RORA sites, all DHS and 10K random genomic locations.
(PDF)

S7 Fig Western blot time-series of CREB and pCREB. Western blot time-series of CREB and pCREB (phosphorylation on Ser 133) in nuclear extracts from WT and *Bmal1*^-/-^ livers (n = 4 individual animals per time point). Loading control shows staining with naphtol blue black.
(PDF)

S8 Fig Proportion of cycling DHSs at BMAL1 bound sites in function of BMAL1 ChIP-seq signal. Top: Percentage of cycling DHSs at BMAL1 bound sites in function of BMAL1 ChIP-seq signal. Bottom: number of sites above a certain BMAL1 signal. Based on S2 Table from [17].
(PDF)

S9 Fig Average profile of DNase I cuts for bound double E-boxes with a spacer of 6 bp. Genomic profiles of DNase I cuts around double E-boxes with a spacer of 6 bp (E1-E2 sp6) at all time points. The analysis is identical to that in Fig 6A. The analysis for ZT6 in *Bmal1*^-/-^ mice is also shown.
(PDF)

S10 Fig Average profile of DNase I cuts for bound double E-boxes with a spacer of 7 bp. Idem as S9 Fig but for double E-boxes with a spacer of 7 bp.
(PDF)

S11 Fig Average profile of DNase I cuts for bound single E-boxes sites. Idem as Fig 6A but selecting BMAL1 bound DHSs containing single E-boxes. Otherwise the analysis is identical to S9 and S10 Figs.
(PDF)

S12 Fig Average profile of DNase I cuts for bound USF1 sites. Idem as Fig 6A, but selecting DHSs bound by USF1 and containing a USF1 motif (E-box).
(PDF)

S13 Fig Average profile of DNase I cuts for bound sites of various TFs. Idem as Fig 6A, but selecting DHSs bound by REV-ERB, HSF1, SREBP and CREB, and containing the corresponding motifs. Here, DHS sites overlapped by a high ChIP-seq signal (Z score > 2) were considered.
(PDF)

S14 Fig ChIP-seq for co-activator or co-repressors at BMAL1 target sites with single or tandem E-boxes. ChIP-seq data from [11] were reanalyzed on BMAL1 ChIP-seq targets [17] overlapping a DHS. CT8 and CT20 time points from [11] are plotted for core clock activators (BMAL1, CLOCK, NPAS2), co-repressors (PER1/2, CRY1/2), and co-activators (CBP, P300). The signal of these different data sets is plotted on DHSs that are also BMAL1 targets, without E-boxes (n = 678), a single E-box (E1 sites, n = 742) or a double E-box (E1E2, n = 217).
(PDF)

S1 Table Quality controls for sequenced libraries. Quality control and mapping statistics for DNase I, Pol II and H3K27ac to the mm9 genome assembly.
(XLSX)

S2 Table All identified DHSs with associated data. The columns contain the genomic location of the DHS and the closest TSS, with the relative and absolute distance between them as well as the TSS name, the strand and the type (i.e. protein coding), the DHS class (TSS, proximal, distal) and quantified signals for DNase I, Pol II and H3K27ac in WT and *Bmal1*^-/-^, at the ZT2, ZT6, ZT10, ZT14, ZT18, ZT22, ZT26 time points as indicated in the columns headers. The two last column provide the gene symbol of the closest TSS and the number of footprints in the DHS.
(XLSX)

S3 Table KEGG and Reactome pathway analysis. KEGG and Reactome Pathway analysis of oscillating genes in mRNA accumulation in WT and *Bmal1*^-/-^ mice. This table includes the genotype (WT or *Bmal1*^-/-^), the p-value of enrichment (hyper geometric test), the number of genes annotated with a specific term in the annotation (term.size), the total number of genes annotated in the query (query.size), the number of genes in the hits belonging to a specific annotation (overlap.size), the recall and the precision, the annotation ID (term.ID), the annotation name (term.name) and the genes names of the hits belonging to a specific annotation (intersection).
(XLSX)

S4 Table Inferred activity of motifs retained by the penalized linear model. Inferred activity (phase and amplitudes) for PWMs (DNA motifs) retained by the penalized generalized linear model using Pol II loadings at TSS and motif content in DHSs within 50kb from the gene TSSs. The consensus sequence, the source of the PWM, the number of targets and the sum of motifs in DHSs are listed.
(XLSX)

S1 File Mixture model for DNase I-seq footprints. (PDF)

S2 File Hetero-tetramer of CLOCK:BMAL1 in.pdb format. (PDB)

S1 Movie Animation of temporal chromatin states at the *Dbp* locus. Dynamics of DNase I, Pol II and H3K27ac at the *Dbp* locus.
(GIF)

S2 Movie Animation of temporal chromatin states at the *Npas2* locus. Dynamics of DNase I, Pol II and H3K27ac at the *Npas2* locus.
(GIF)

S3 Movie 3D structure of the Hetero-tetramer of CLOCK:BMAL1 (sp6). (MOV)

S4 Movie 3D structure of the Hetero-tetramer of CLOCK:BMAL1 (sp7). (MOV)

## Abbreviations

ChIP-seq: chromatin immunoprecipitation followed by DNA sequencing
DHS: DNase I hypersensitive site
eRNA: enhancer RNA
FDR: false discovery rate
FOX: forkhead box
GC: glucocorticoid hormone
GR: glucocorticoid receptor
HSF1: heat shock transcription factor 1
LD: light–dark
PDB: Protein Data Bank
Pol II: RNA polymerase II
RRE: ROR response element
TF: transcription factor
TSS: transcription start site
WT: wild type
ZT: zeitgeber time
