# Supplementary figures and images for: Transcriptional regulatory logic of the diurnal cycle in the mouse liver
Source: PLoS Biol. 2017 Apr 17;15(4):e2001069. doi: 10.1371/journal.pbio.2001069 (PMC5393560; doi:10.1371/journal.pbio.2001069)

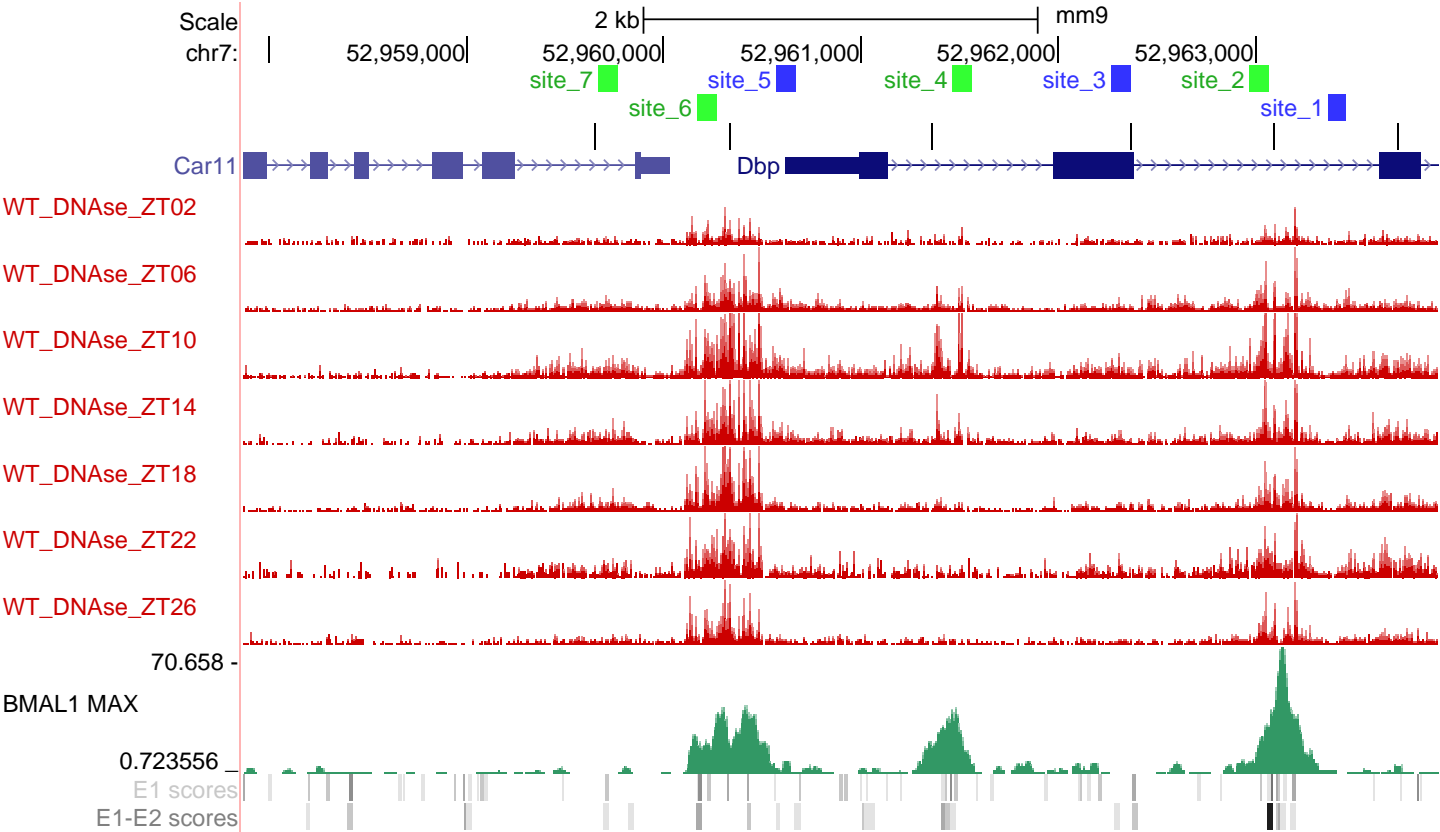

Supplement: S1 Fig — Measured DNase I-seq signals near the Dbp gene, compared with previously reported DHSs in a reference study [30] (marked site_1 to site_7). [30] found seven hypersensitive sites while we detected six DHSs using our peak calling at compatible locations (black marks). Moreover, [30] reported high (sites 2, 4, 6, and 7, in green), or lower (sites 1, 3 and 5 in blue), amplitudes in rhythmic DNase I digestion efficiency, consistent with the DNase I-seq signals (visual inspection). Sites 2, 4, and 7 contain E-boxes that are binding sites for CLOCK and BMAL1. Locations of BMAL1 ChIP-seq signals (bottom track) [17] clearly overlaps strongest DNase I peaks. DNase I browser tracks are normalized to the total read count here. (PDF) [file pbio.2001069.s001.pdf]

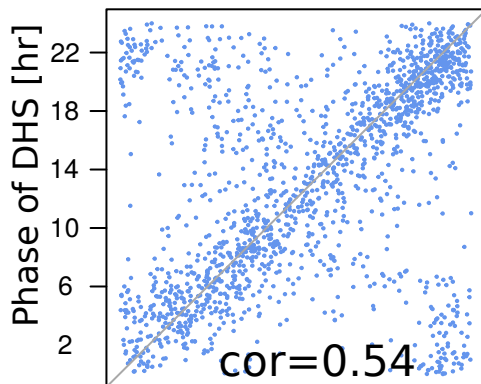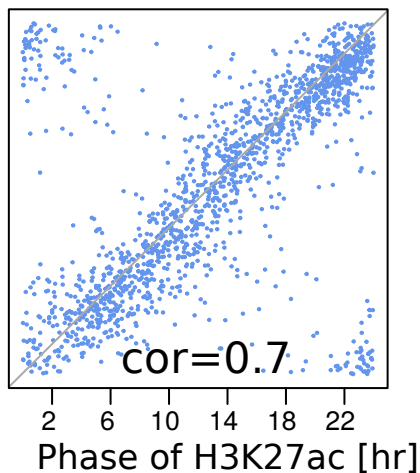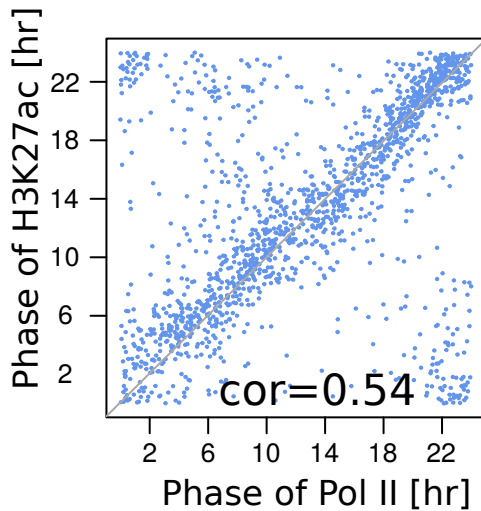

Supplement: S3 Fig — Similarly to Fig 3D, high correlations and no phase shifts can still be observed outside of actively transcribed regions, demonstrating that this relationship is not only linked to active transcription. (PDF) [file pbio.2001069.s003.pdf]

A

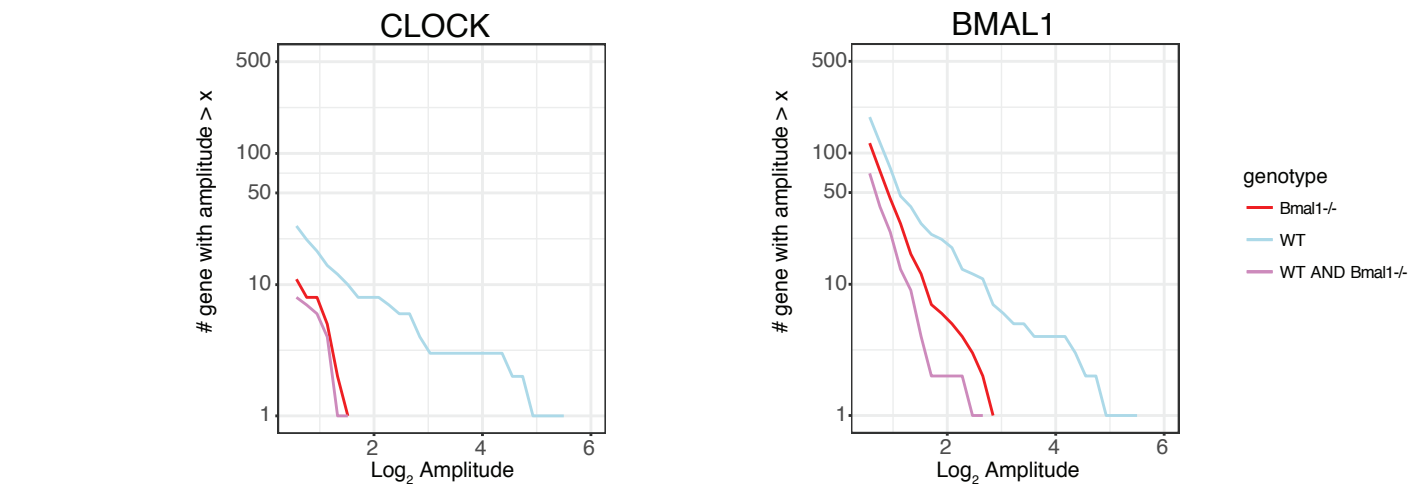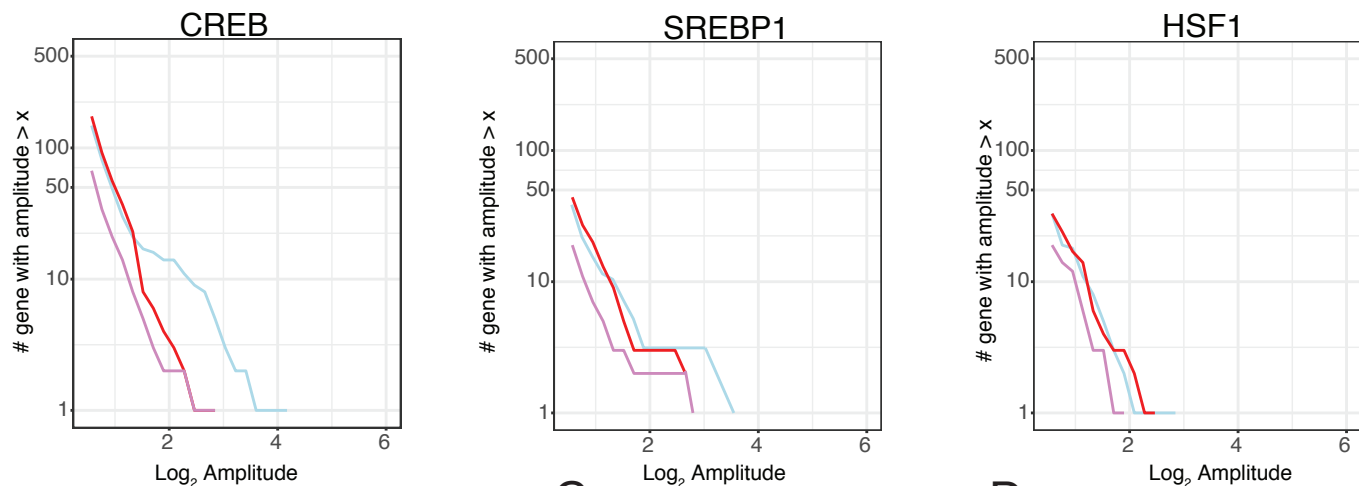

B

Co-occurrence of Motifs in Oscillating TSSs

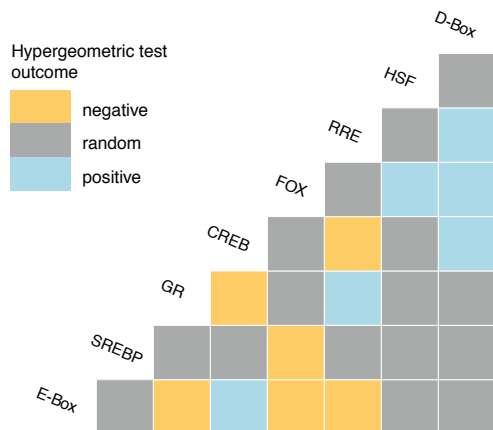

C

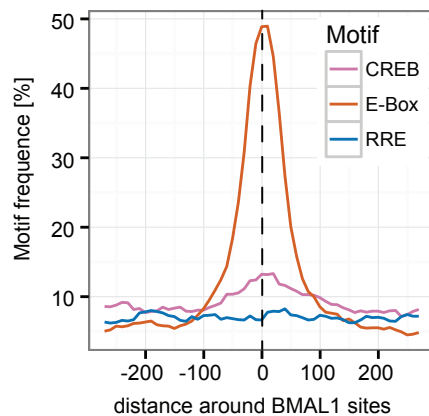

D

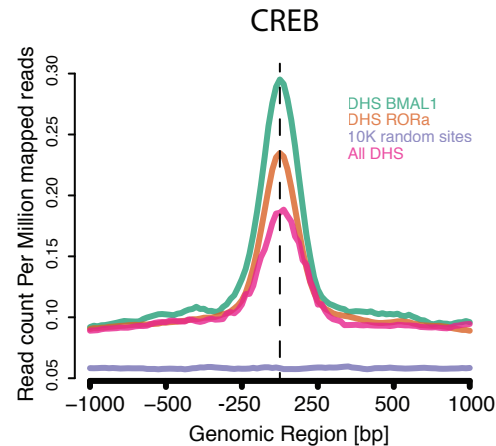

Supplement: S6 Fig — A. Cumulative count of genes with oscillating mRNA accumulation (selected with p < 0.05, harmonic regression) in Bmal1-/- and WT mice, with log2 amplitude greater than the values on the x-axis. The different panels show genes bound by core clock (CLOCK, BMAL1), nutrient-related (CREB and SREBP1) and systemic signal (HSF1) TFs. B. Co-occurrence of DNA motifs on TSSs with cycling DNase I, H3K27ac and Pol II (Fisher’s combined p-value below 0.05). Combinations between DNA motifs for the core clock (E-box, D-box, RRE), the feeding-fasting cycle (FOX, CREB, SREB) and response to systemic cues (GR, HSF) are shown (hypergeometric test, p<0.05). Positive associations are shown in blue and negative associations in orange. C. Motif analysis around BMAL1 ChIP-seq sites [17] for CREB, E-Box and RRE weight matrices (matches use p < 0.001, Oprof in [23]). This motif analysis was performed with a sliding window of 60bp and a 10 bp shift using Oprof from the Signal Search Analysis server. D. ChIP-seq signal for CREB (Fasted conditions, from [63] around BMAL1 sites, RORA sites, all DHS and 10K random genomic locations. (PDF) [file pbio.2001069.s006.pdf]

**A****Nuclear extract  
CREB****WT*****Bmal1*<sup>-/-</sup>**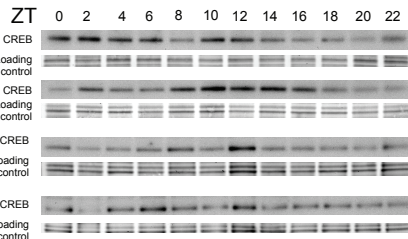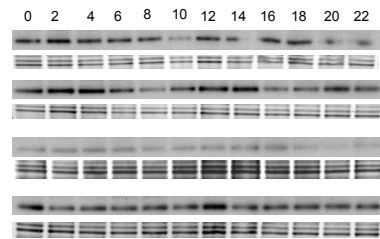**Nuclear extract  
P-CREB****WT*****Bmal1*<sup>-/-</sup>**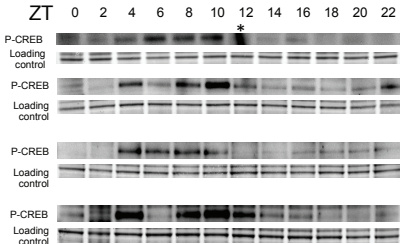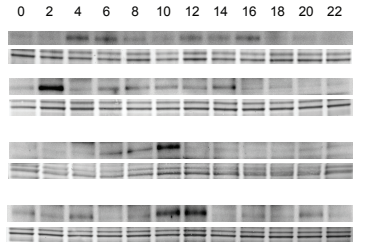

\* Unspecific signal

Supplement: S7 Fig — Western blot time-series of CREB and pCREB (phosphorylation on Ser 133) in nuclear extracts from WT and Bmal1-/- livers (n = 4 individual animals per time point). Loading control shows staining with naphtol blue black. (PDF) [file pbio.2001069.s007.pdf]

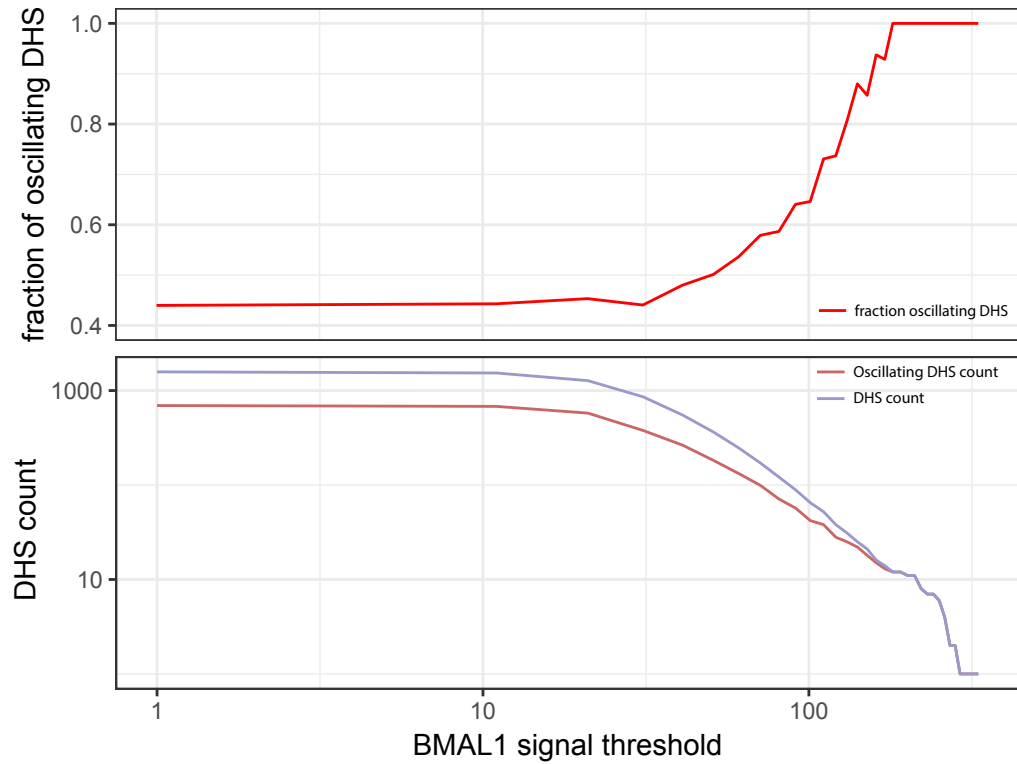

Supplement: S8 Fig — Top: Percentage of cycling DHSs at BMAL1 bound sites in function of BMAL1 ChIP-seq signal. Bottom: number of sites above a certain BMAL1 signal. Based on S2 Table from [17]. (PDF) [file pbio.2001069.s008.pdf]

## BMAL E1-E2 sp6

ZT2

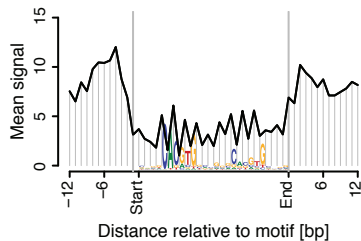

ZT6

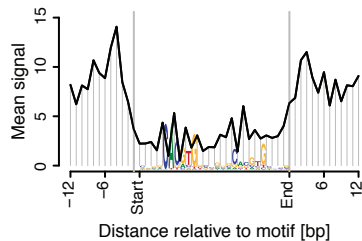

ZT10

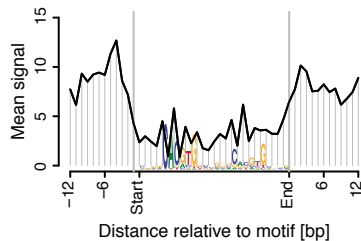

ZT14

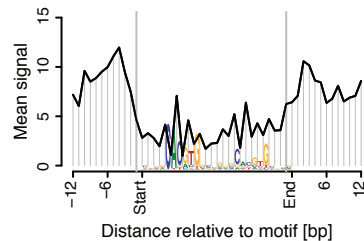

ZT18

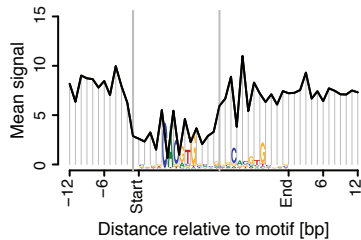

ZT22

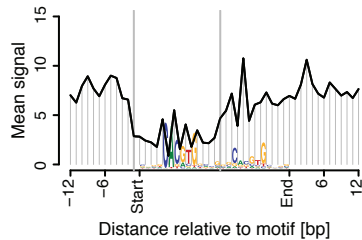

ZT26

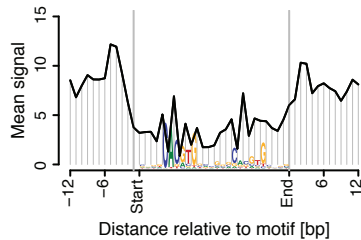

ZT6 *Bmal1*<sup>-/-</sup>

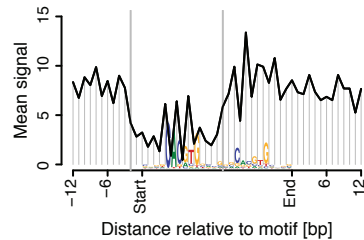

Supplement: S9 Fig — Genomic profiles of DNase I cuts around double E-boxes with a spacer of 6 bp (E1-E2 sp6) at all time points. The analysis is identical to that in Fig 6A. The analysis for ZT6 in Bmal1-/- mice is also shown. (PDF) [file pbio.2001069.s009.pdf]

## BMAL E1-E2 sp7

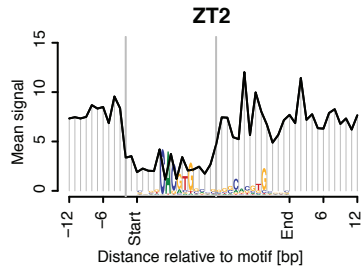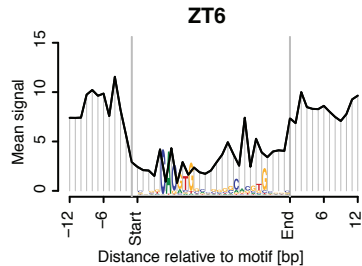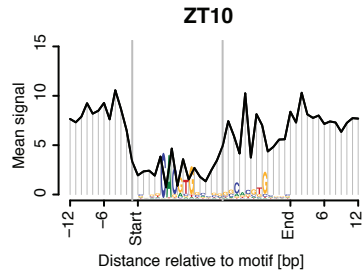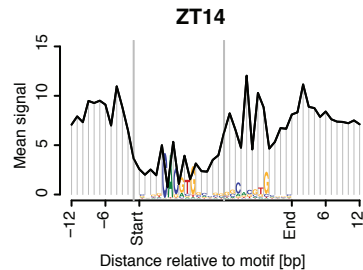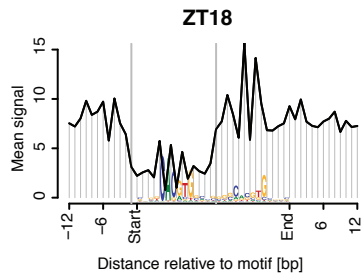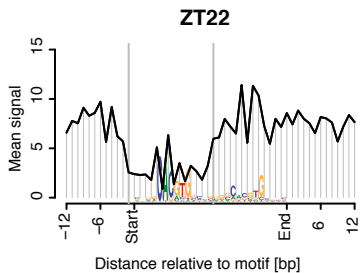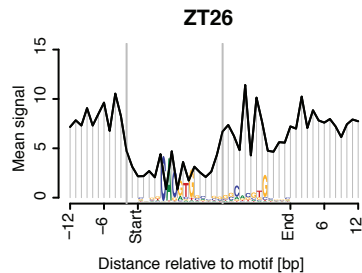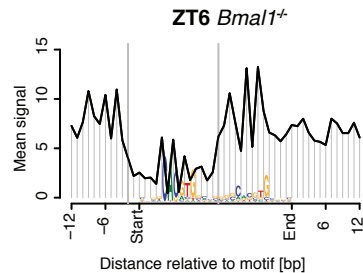

Supplement: S10 Fig — Idem as S9 Fig but for double E-boxes with a spacer of 7 bp. (PDF) [file pbio.2001069.s010.pdf]

## BMAL Single E-box

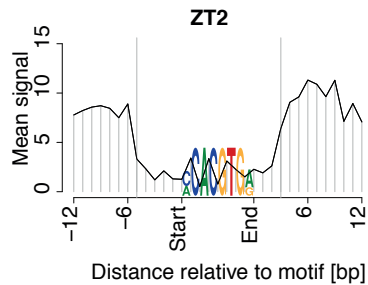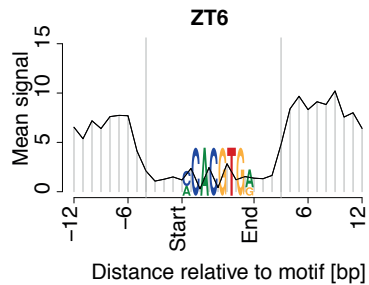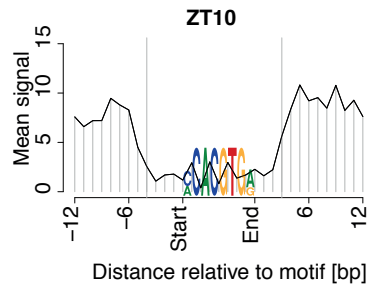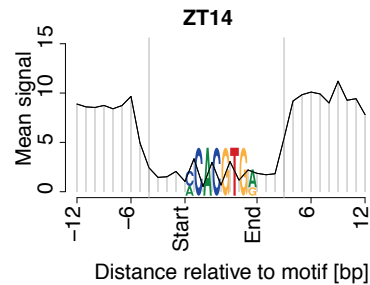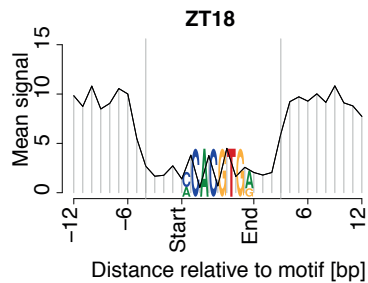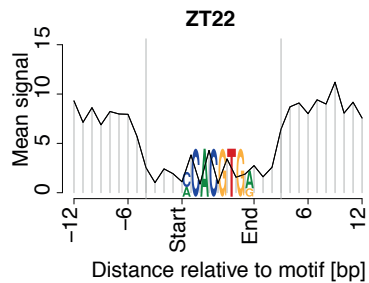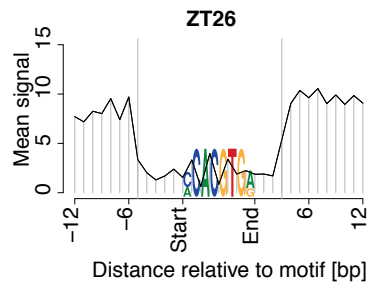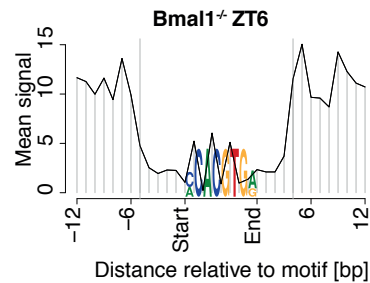

Supplement: S11 Fig — Idem as Fig 6A but selecting BMAL1 bound DHSs containing single E-boxes. Otherwise the analysis is identical to S9 and S10 Figs. (PDF) [file pbio.2001069.s011.pdf]

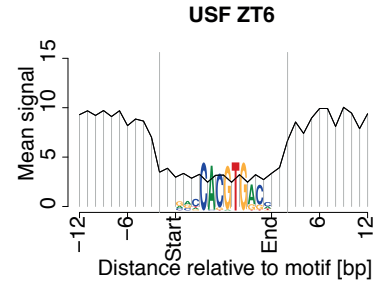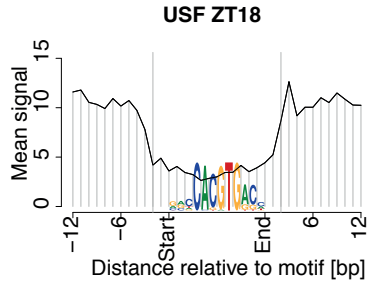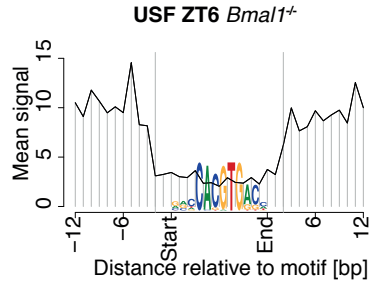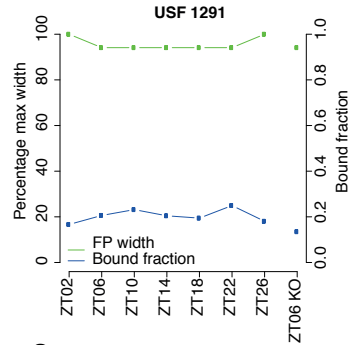

Supplement: S12 Fig — Idem as Fig 6A, but selecting DHSs bound by USF1 and containing a USF1 motif (E-box). (PDF) [file pbio.2001069.s012.pdf]

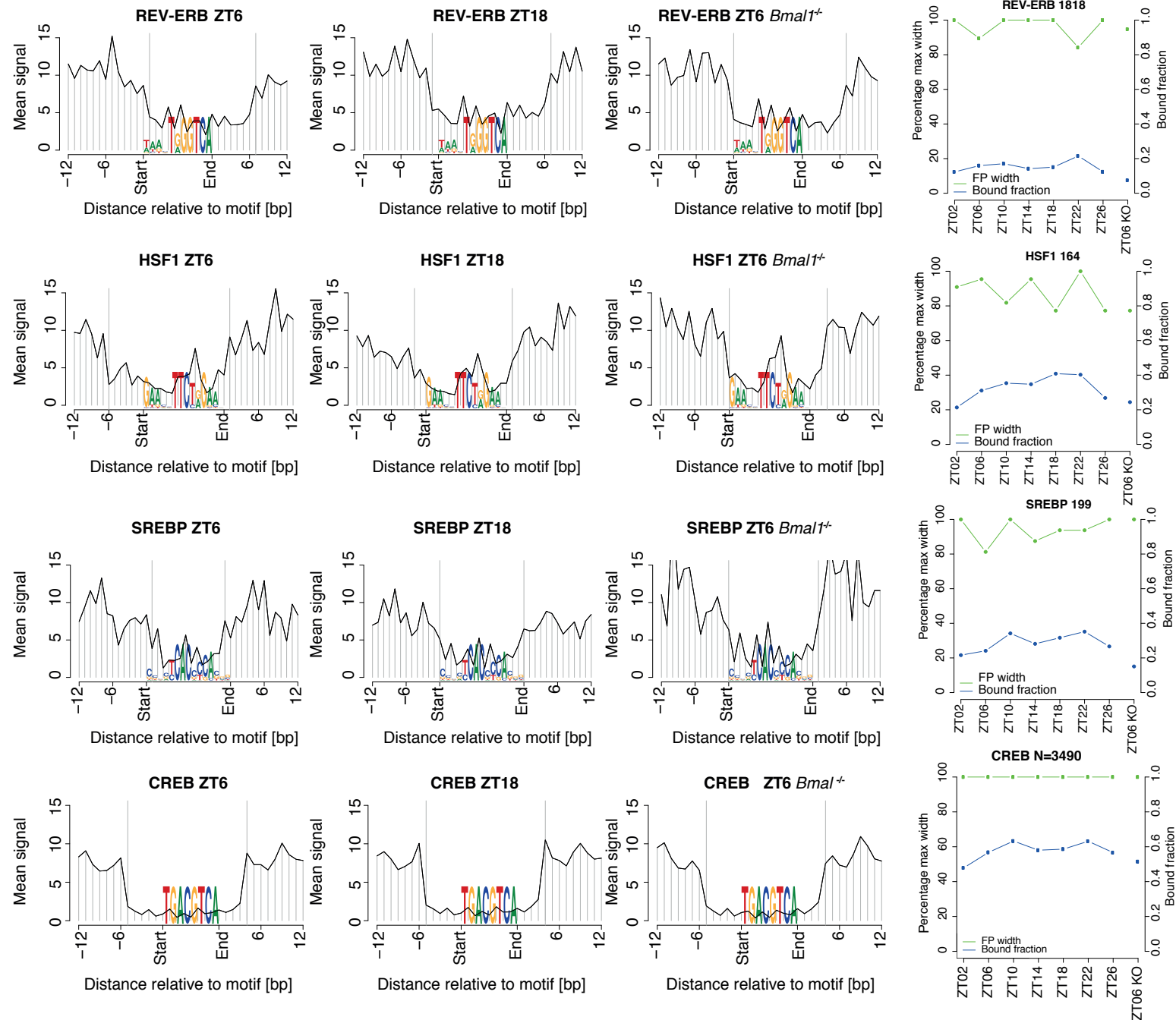

Supplement: S13 Fig — Idem as Fig 6A, but selecting DHSs bound by REV-ERB, HSF1, SREBP and CREB, and containing the corresponding motifs. Here, DHS sites overlapped by a high ChIP-seq signal (Z score > 2) were considered. (PDF) [file pbio.2001069.s013.pdf]

Read count Per Million mapped reads

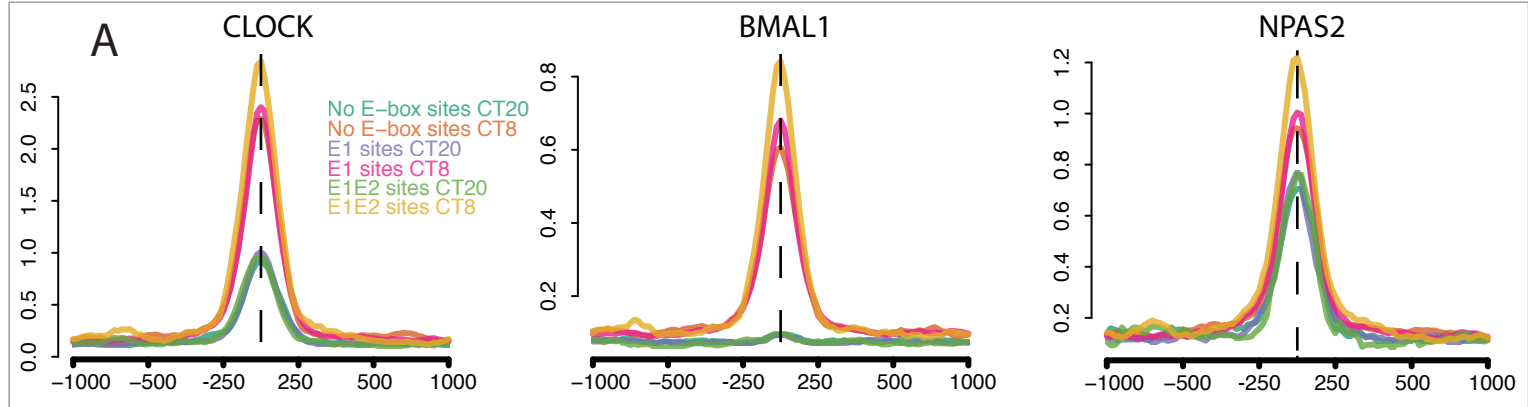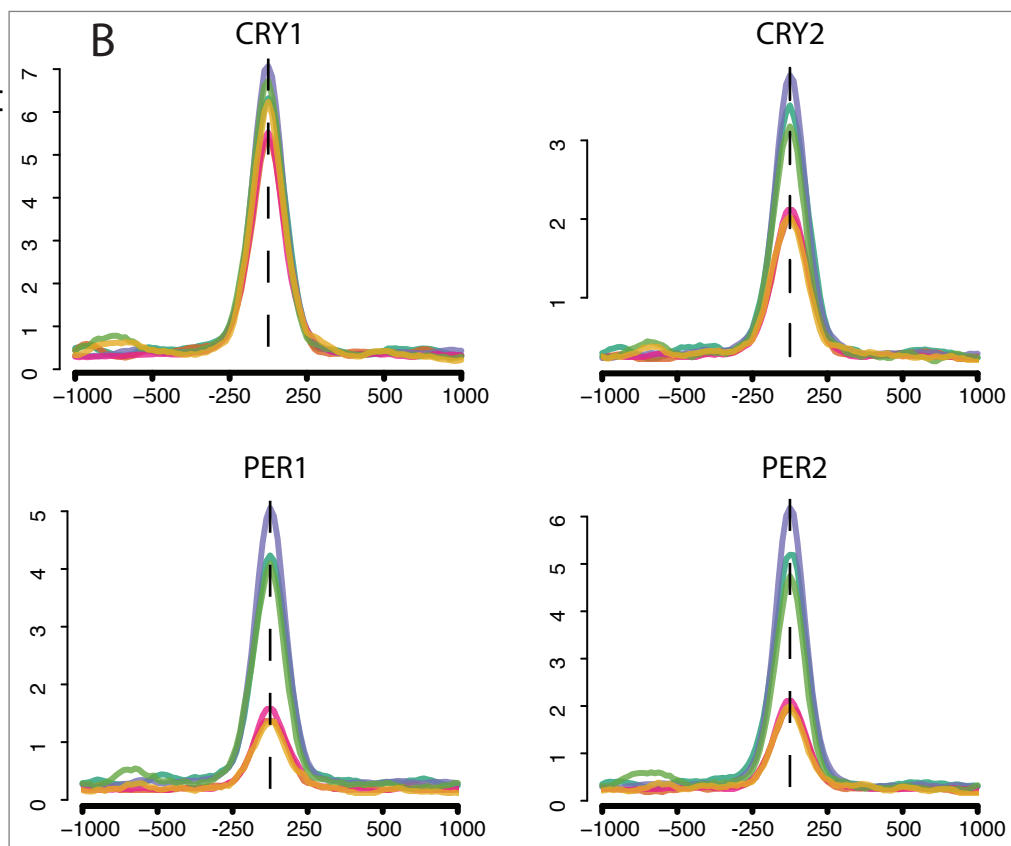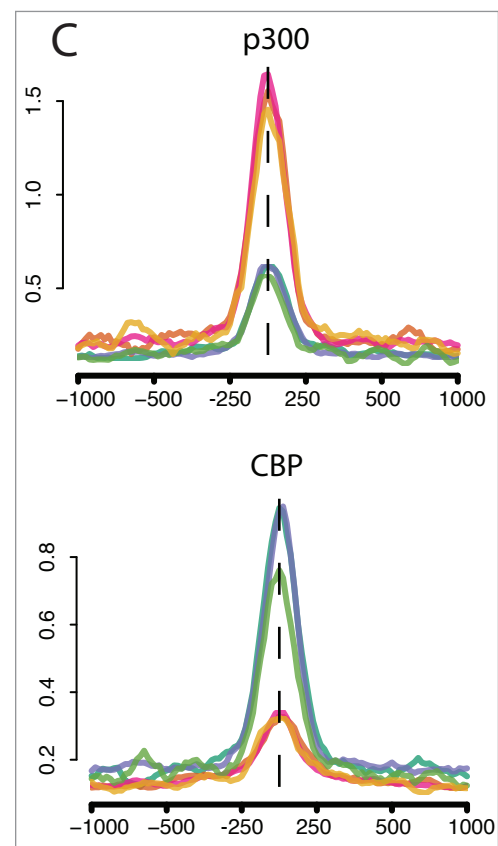

Genomic Region around Bmal1 sites [bp]

Supplement: S14 Fig — ChIP-seq data from [11] were reanalyzed on BMAL1 ChIP-seq targets [17] overlapping a DHS. CT8 and CT20 time points from [11] are plotted for core clock activators (BMAL1, CLOCK, NPAS2), co-repressors (PER1/2, CRY1/2), and co-activators (CBP, P300). The signal of these different data sets is plotted on DHSs that are also BMAL1 targets, without E-boxes (n = 678), a single E-box (E1 sites, n = 742) or a double E-box (E1E2, n = 217). (PDF) [file pbio.2001069.s014.pdf]

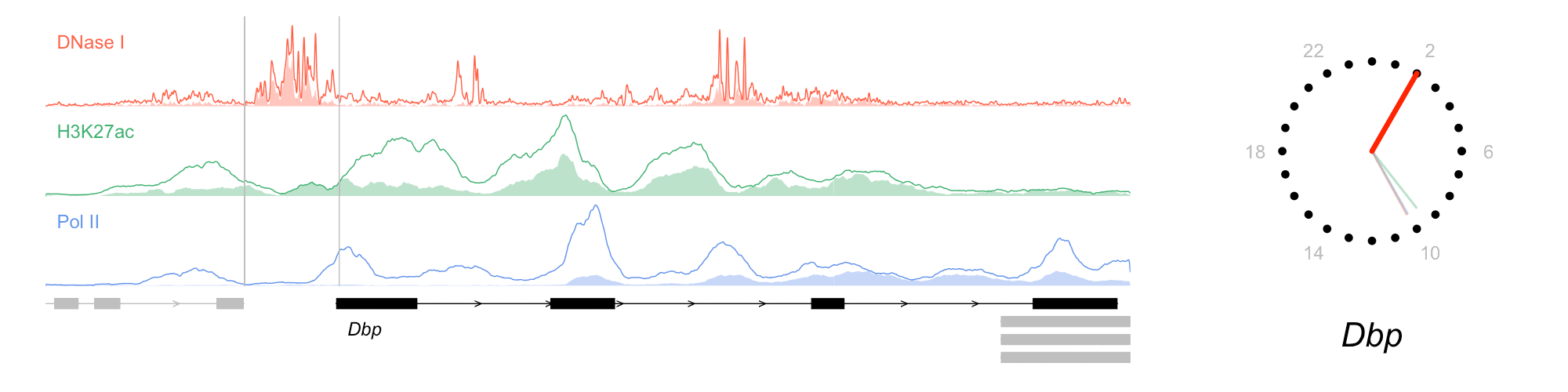

Supplement: S1 Movie — Dynamics of DNase I, Pol II and H3K27ac at the Dbp locus. (GIF) [file pbio.2001069.s021.gif]

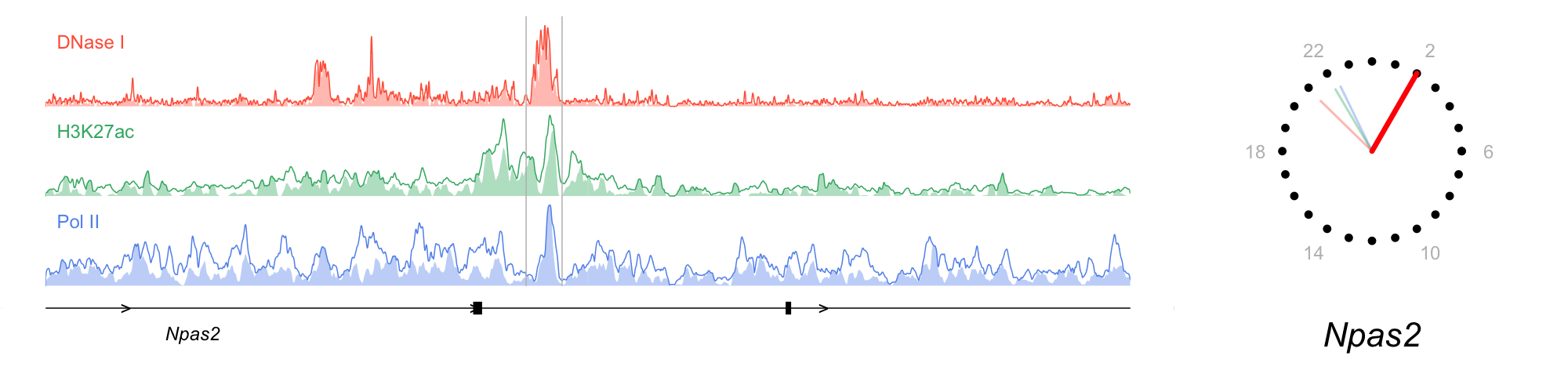

Supplement: S2 Movie — Dynamics of DNase I, Pol II and H3K27ac at the Npas2 locus. (GIF) [file pbio.2001069.s022.gif]
