# Supplementary material for: Transcriptional regulatory logic of the diurnal cycle in the mouse liver
Source: PLoS Biol. 2017 Apr 17;15(4):e2001069. doi: 10.1371/journal.pbio.2001069 (PMC5393560; doi:10.1371/journal.pbio.2001069)
Supplement: S2 Fig — A. Distribution of distances between DHSs and nearest active TSSs. We observe a bimodal distribution, with a first mode corresponding to DHSs in promoter regions (centered on 100 bp from the TSS) and a second mode centered on 10 kb from TSSs. B. Repartition of DHSs within three classes depending on their distance from the nearest TSS: 47% are more than 10 kb from a TSS and are classified as distal, 28% are between 1 kb and 10 kb away and are classified as proximal, and DHSs located 1 kb or less from a TSS represent 24% of all sites. C-D. Pol II, DHS and H3K27ac signals around TSSs and distal DHSs (averages over all sites). Profiles were normalized so that the maximum around the TSS is 100%. E. DNase I signals (all time points are merged in the ZT All track) near the Albumin gene. Footprint detected using the Wellington algorithm are shown below the detected DHS sites. The promoter region is enlarged at the bottom, showing that the wide footprint detected in our data corresponds to previously established transcription factor binding sites (the colored boxed indicate protein complexes previously identified in [47]). Many sensitive regions locate din the gene body do not display footprints, probably due to high transcription of Alb in the liver. (PDF) [file pbio.2001069.s002.pdf]

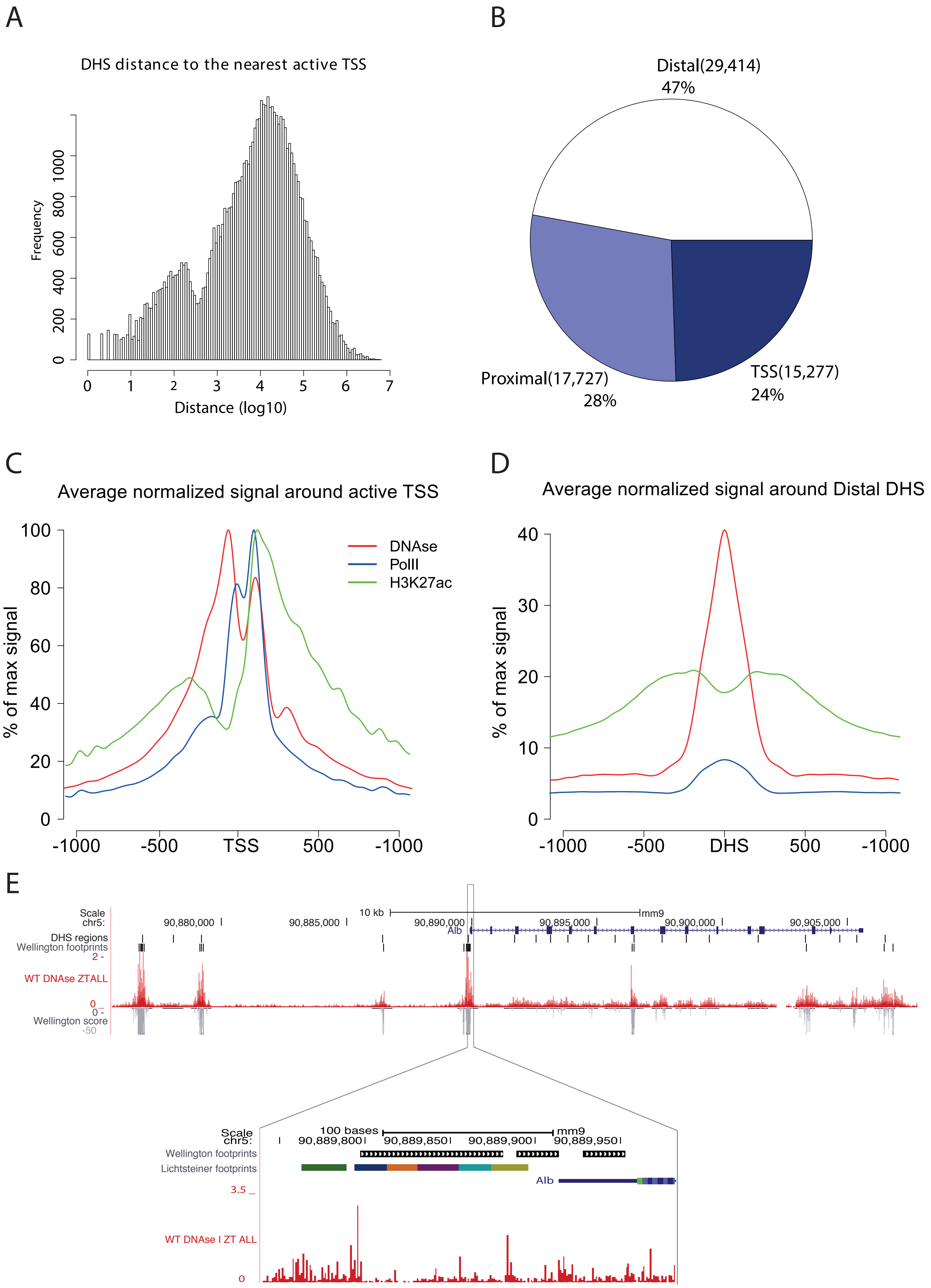
A
DHS distance to the nearest active TSS
Frequency
0
200
400
600
800
1000
0 1 2 3 4 5 6 7
Distance (log10)
B
Distal(29,414)
47%
Proximal(17,727)
28%
TSS(15,277)
24%
C
Average normalized signal around active TSS
% of max signal
0
20
40
60
80
100
DNAse
PolII
H3K27ac
-1000 -500 TSS 500 -1000
D
Average normalized signal around Distal DHS
% of max signal
0
10
20
30
40
-1000 -500 DHS 500 -1000
E
Scale
chr5:
10 kb
mm9
90,880,000
90,885,000
90,890,000
90,895,000
90,900,000
90,905,000
Alb
DHS regions
Wellington footprints
2 -
WT DNAse ZTALL
0 _
0 -
Wellington score
-50 _
Scale
chr5:
100 bases
mm9
90,889,800
90,889,850
90,889,900
90,889,950
Wellington footprints
Lichtsteiner footprints
Alb
3.5 _
WT DNAse I ZT ALL
0
