# Supplementary material for: Transcriptional regulatory logic of the diurnal cycle in the mouse liver
Source: PLoS Biol. 2017 Apr 17;15(4):e2001069. doi: 10.1371/journal.pbio.2001069 (PMC5393560; doi:10.1371/journal.pbio.2001069)
Supplement: S4 Fig — A. Number of oscillating genes in WT and in Bmal1-/- mice using Pol II loadings at TSSs and mRNA. B. Cumulative count of oscillating genes (selected with p < 0.05, harmonic regression) in Bmal1-/- and WT mice with log2 amplitude greater or equal than the values on the x-axis. Both Pol II loadings at TSSs and mRNA are shown. Values below 0.5 on the x-axis are not shown. C. Peak times (ZT times) of genes oscillating in WT and in Bmal1-/- using Pol II loadings at TSS. D. Idem using mRNA accumulation profiles. (PDF) [file pbio.2001069.s004.pdf]

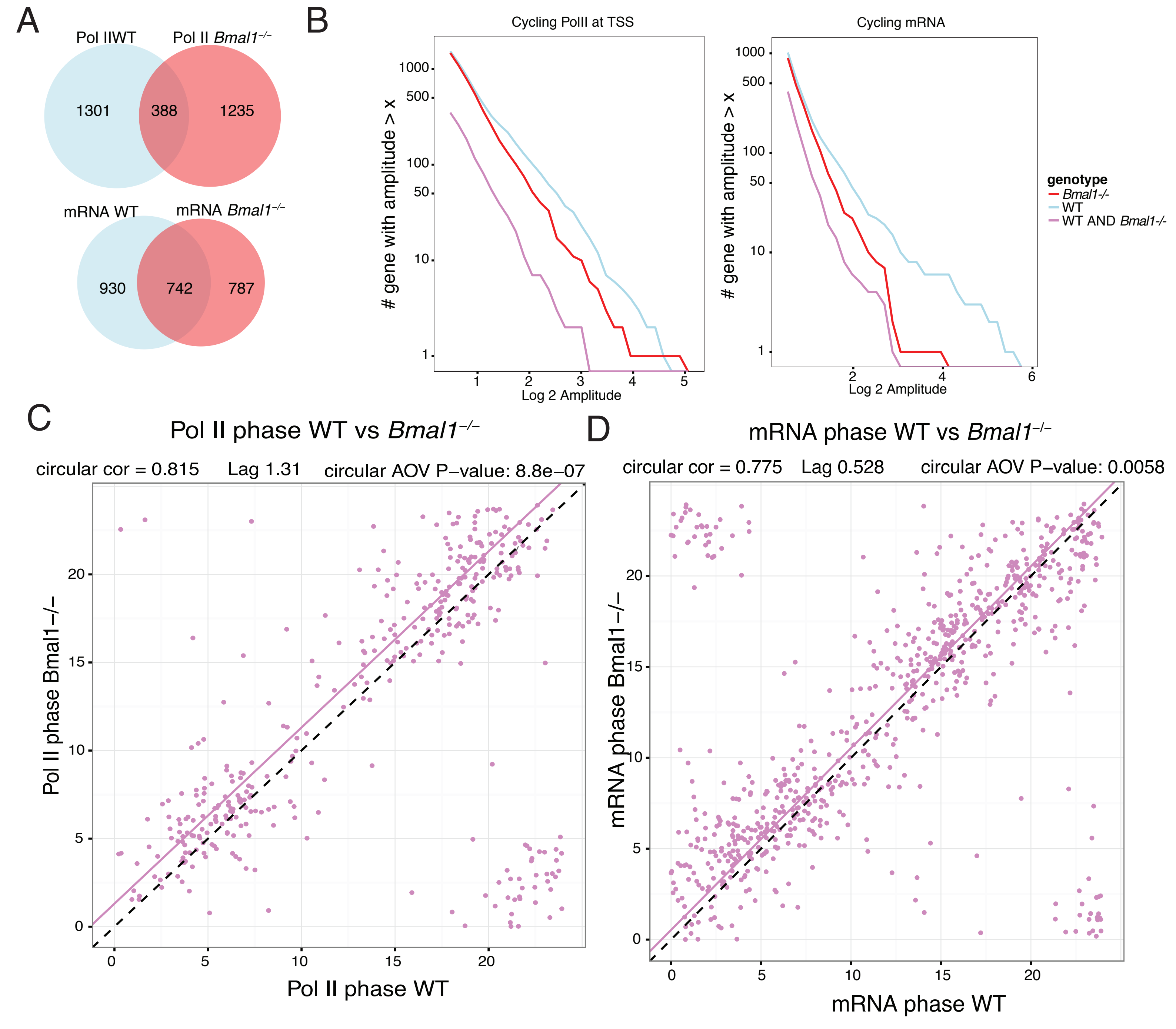
A
Pol IIWT
Pol II Bmal1−/−
1301
388
1235
mRNA WT
mRNA Bmal1−/−
930
742
787
B
Cycling PolII at TSS
gene with amplitude > x
Log 2 Amplitude
Cycling mRNA
genotype
Bmal1-/-
WT
WT AND Bmal1-/-
C
Pol II phase WT vs Bmal1−/−
circular cor = 0.815
Lag 1.31
circular AOV P−value: 8.8e−07
Pol II phase Bmal1−/−
Pol II phase WT
D
mRNA phase WT vs Bmal1−/−
circular cor = 0.775
Lag 0.528
circular AOV P−value: 0.0058
mRNA phase Bmal1−/−
mRNA phase WT
