# Supplementary material for: Transcriptional regulatory logic of the diurnal cycle in the mouse liver
Source: PLoS Biol. 2017 Apr 17;15(4):e2001069. doi: 10.1371/journal.pbio.2001069 (PMC5393560; doi:10.1371/journal.pbio.2001069)
Supplement: S5 Fig — A. Boxplot of log2 mRNA amplitude of genes from significantly enriched Kegg or Reactome pathway in WT and in Bmal1-/- genotypes. These pathways were retrieved using g:Profiler with p<0.1 in one or both genotype. Boxplots were generated with the amplitude of each oscillating genes annotated with a specific pathway. Genes used for each annotation are reported in S3 Table. B. Phase and amplitude of mRNAs for significantly enriched annotations in WT and in Bmal1-/- such as circadian rhythm, cholesterol biosynthesis, bile secretion, triglyceride biosynthesis, ribosome biogenesis and M/G1 transition associated genes, reported in a polar scatter plot. Green arrows represent mRNA with higher amplitudes in Bmal1-/- mice, while red arrows represent genes that loose rhythmicity in the knockout. (PDF) [file pbio.2001069.s005.pdf]

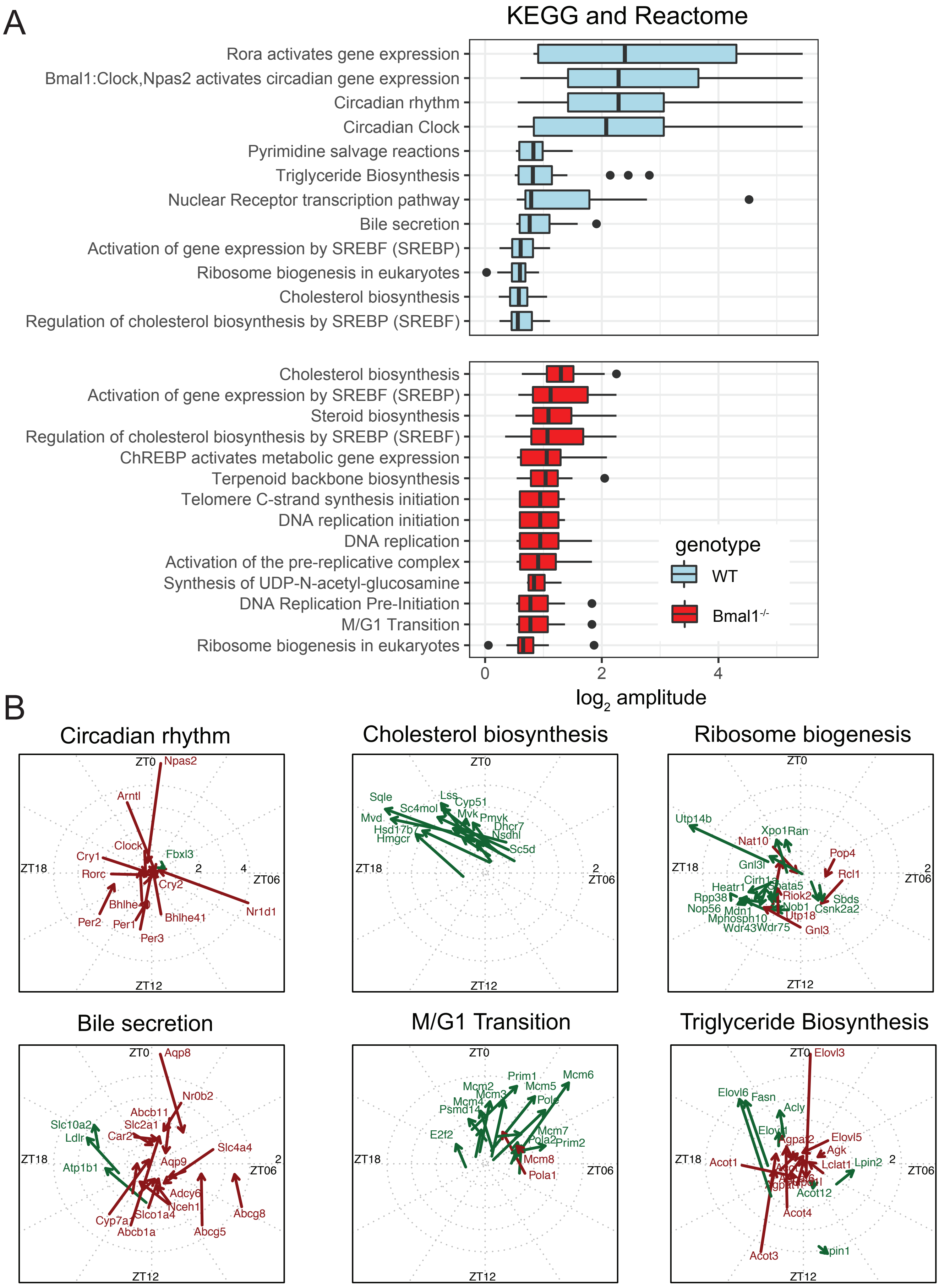

A
KEGG and Reactome
Rora activates gene expression
Bmal1:Clock,Npas2 activates circadian gene expression
Circadian rhythm
Circadian Clock
Pyrimidine salvage reactions
Triglyceride Biosynthesis
Nuclear Receptor transcription pathway
Bile secretion
Activation of gene expression by SREBF (SREBP)
Ribosome biogenesis in eukaryotes
Cholesterol biosynthesis
Regulation of cholesterol biosynthesis by SREBP (SREBF)
Cholesterol biosynthesis
Activation of gene expression by SREBF (SREBP)
Steroid biosynthesis
Regulation of cholesterol biosynthesis by SREBP (SREBF)
ChREBP activates metabolic gene expression
Terpenoid backbone biosynthesis
Telomere C-strand synthesis initiation
DNA replication initiation
DNA replication
Activation of the pre-replicative complex
Synthesis of UDP-N-acetyl-glucosamine
DNA Replication Pre-Initiation
M/G1 Transition
Ribosome biogenesis in eukaryotes
genotype
WT
Bmal1-/-
0
2
4
log2 amplitude
B
Circadian rhythm
Cholesterol biosynthesis
Ribosome biogenesis
Bile secretion
M/G1 Transition
Triglyceride Biosynthesis
ZT0
ZT06
ZT12
ZT18
Npas2
Arntl
Clock
Cry1
Rorc
Fbxl3
Cry2
Nr1d1
Bhlhe41
Per1
Per2
Per3
Sqle
Lss
Sc4mol
Cyp51
Mvd
Mvk
Pmvk
Hsd17b7
Hmgcr
Dhcr7
Nsdhl
Sc5d
Utp14b
Xpo1Ran
Nat10
Pop4
Rcl1
Gnl3l
Cirh1a
Heatr1
Rpp38
Nop56
Mdn1
Mphosph10
Wdr43Wdr75
Riok2
Nob1
Utp18
Sbds
Csnk2a2
Gnl3
Aqp8
Nr0b2
Abcb11
Slc2a1
Slc10a2
Ldlr
Car2
Atp1b1
Aqp9
Slc4a4
Adcy6
Nceh1
Cyp7a1
Slco1a4
Abcb1a
Abcg5
Abcg8
Prim1
Mcm6
Mcm2
Mcm5
Mcm3
Mcm4
Psmd14
Pole
E2f2
Mcm7
Pola2
Prim2
Mcm8
Pola1
Elovl3
Elovl6
Fasn
Acly
Elovl5
Agk
Acot1
Lclat1
Lpin2
Acot12
Acot4
Acot3
