# Supplementary material for: Transcriptional regulatory logic of the diurnal cycle in the mouse liver
Source: PLoS Biol. 2017 Apr 17;15(4):e2001069. doi: 10.1371/journal.pbio.2001069 (PMC5393560; doi:10.1371/journal.pbio.2001069)
Supplement: S1 File — (PDF) [file pbio.2001069.s019.pdf]

1 Mixture model for DNase I data

We assume to have DNase I cuts $n_{ri} \in \mathbb{N}$ for regions $r \in \{1, \ldots, R\}$ at positions $i \in \{1, \ldots, L\}$, where R is the number of regions and L the length of the regions (all assumed to be of the same length). Indices i_L and i_R refer to boundary positions (the left and right boundaries of a protected sequence motif) and we assume that the cuts are all aligned with respect to some ankor, for example the position of a sequence motif reflecting the specificity of a known protein-DNA interaction. We define $J = \{i_L, \ldots, i_R\}$ and $I = \{1, \ldots, L\} \backslash J$.

The goal is to calibrate a mixture model from the data, such that we can learn which sites are bound (showing footprints), and which are not. While similar approaches have been proposed [Pique-Regi et al., 2010], we are interested in additionally learning the optimal boundary positions, such that we can detect if a footprint changes shape (in our case its width) in different conditions (for example at different time points). To make the model tractable, we make the simplifying assumption that the shape of a footprint can be approximated by a rectangular shape, showing on average less counts in the protected regions. While it was shown previously that the signal within the protected region can be nonuniform for some factors [Neph et al., 2012], the rectangular model is a simple generic model that captures the essential properties of DNase I signals around bound sites in many cases. Specifically, we express the probability (likelihood) of the measured cuts $\vec{n} = (n_1, \ldots, n_L)$ (here for a single region or one row in the matrix n_{ri}) as a product of independent Poisson variables with a common mean λ when the site is not bound

$$P_1(\vec{n}|\vec{\lambda}) = \prod_{i=1}^{L} Pois(n_i|\lambda) = \mathcal{M}(\vec{n}|\vec{p}, N) Pois(N|\Lambda) \equiv Q_1(\vec{n}; \Lambda) \quad , \tag{1}$$

where we used the property that products of independent Poisson variable can be factored into a multinomial ($\mathcal{M}$) and one Poisson distribution, and defined $N = \sum_i n_i$, $\Lambda = \sum_i \lambda_i = L\lambda$, $p_i = \frac{1}{L}$. The notation Q_1 emphasises that this is now a function of Λ.

For the second (bound) model we assume two distinct means λ_I for unprotected, and λ_J for protected sites, representing the average number of cuts outside ($i \in I$), and inside ($j \in J$) the footprinted region, respectively. This leads to

$$\begin{aligned} P_2(\vec{n}|\lambda_I, \lambda_J, i_L, i_R) &= \prod_{i \in I} P(n_i|\lambda_I) \prod_{j \in J} P(n_j|\lambda_J) & (2) \\ &= \mathcal{M}(\vec{n}|\vec{q}, N) Pois(N|\Lambda) \equiv Q_2(\vec{n}; i_L, i_R, q_J, \Lambda) \quad , & (3) \end{aligned}$$

where here $\Lambda = \sum_i \lambda_i = L_1\lambda_I + L_2\lambda_J$ with $L_1 = |I|$, $L_2 = |J|$, $q_i = \lambda_I/\Lambda \equiv q_I$ for $i \in I$ and $q_i = \lambda_J/\Lambda \equiv q_J$ for $i \in J$. The notation Q_2 shows the dependencies in the new variables.

We then marginalize the probabilities over the unknown Λ (using an improper flat prior, such that $\int_0^\infty Pois(N|\Lambda)d\Lambda = P(N) = 1$, and thus equivalent to making no assumption on the total number of cuts). After some straightforward algebra, this leads to

$$F_1(\vec{n}) = \int_0^\infty d\Lambda Q_1(\vec{n};\Lambda) = \frac{N!}{\prod_i (n_i!)}\, L^{-N} \tag{4}$$

where $N = \sum_i n_i$, and

$$\begin{aligned} F_2(\vec{n}|i_L, i_R) &= L \int_0^{1/L} dq_J \int d\Lambda\, Q_2(\vec{n};i_L, i_R, q_J, \Lambda) & (5) \\ &= \frac{1}{N+1} \frac{N_1!}{\prod_{i\in I}(n_i!)} \frac{N_2!}{\prod_{j\in J}(n_j!)}\, L_1^{-N_1} L_2^{-N_2} \frac{1}{r}\, I_r(N_2+1, N_1+1) & (6) \end{aligned}$$

where $N_1 = \sum_{i\in I} n_i$, $N_2 = \sum_{j\in J} n_j$, $r = \frac{L_2}{L}$, and $I_r(\alpha, \beta)$ is the regularized incomplete Beta function ($I_1(\alpha, \beta) = 1$) that comes form the q_J integral. Note that since the same improper (not normalized) prior on Λ is used for both models, this does not pose any difficulties. The upper integration bound on the q_J integral uses the assumption that $q_J \le q_I \le 1/L$, reflecting that the probability of cuts is reduced inside J due to protection from the bound protein.

We can now formulate the mixture model by introducing a global probability q (to be estimated) to be in the bound state, such that

$$P(\vec{n}|i_L, i_R, q) = (1-q)F_1(\vec{n}) + q\, F_2(\vec{n}|i_L, i_R) \quad , \tag{7}$$

or since q is assumed to be common to all regions

$$P(\{n_{ri}\}|i_L, i_R, q) = \prod_r P(n_{r\bullet}|i_L, i_R, q) \quad . \tag{8}$$

Finally we can marginalize over q to obtain the likelihood of the whole data with respect to the indices I:

$$P(\{n_{ri}\}|i_L, i_R) = \int_0^1 dq\, P(\{n_{ri}\}|i_L, i_R, q) \quad , \tag{9}$$

where we have assumed a uniform prior on q. The interesting aspect is that this likelihood calculation requires only doing a one-dimensional numerical integral, and one can then maximize with respect to the discrete indices i_L and i_R to find the optimal boundaries. Once these have been found, it is straightforward to estimate the optimal q using Eq. 8, and also to assign posterior probabilities to each region for each of the two models using Eqs. 4 and 6.

References

[Neph et al., 2012] Neph, S., Vierstra, J., Stergachis, A. B., Reynolds, A. P., Haugen, E., Vernot, B., Thurman, R. E., John, S., Sandstrom, R., Johnson, A. K., Maurano, M. T., Humbert, R., Rynes, E., Wang, H., Vong, S., Lee, K., Bates, D., Diegel, M.,

Roach, V., Dunn, D., Neri, J., Schafer, A., Hansen, R. S., Kutyavin, T., Giste, E., Weaver, M., Canfield, T., Sabo, P., Zhang, M., Balasundaram, G., Byron, R., MacCoss, M. J., Akey, J. M., Bender, M. A., Groudine, M., Kaul, R., and Stamatoyannopoulos, J. A. (2012). An expansive human regulatory lexicon encoded in transcription factor footprints. *Nature*, 489(7414):83–90.

[Pique-Regi et al., 2010] Pique-Regi, R., Degner, J. F., Pai, A. A., Gaffney, D. J., Gilad, Y., and Pritchard, J. K. (2010). Accurate inference of transcription factor binding from DNA sequence and chromatin accessibility data. *Genome Research*, 21(3):447–455.
